# Vitamin D and Pancreatic Ductal Adenocarcinoma—A Review of a Complicated Relationship

**DOI:** 10.3390/nu16234085

**Published:** 2024-11-27

**Authors:** Iustina Grosu, Alexandru Constantinescu, Mihaela Daniela Balta, Ovidiu Băjenaru, Cătălina Nuța, Christopher Pavel, Vasile Sandru, Mihai Munteanu, Octavian Andronic

**Affiliations:** 1 Endocrinology Department, “Elias” University Emergency Hospital, 011461 Bucharest, Romania; iustina.grosu@rez.umfcd.ro; 2 Faculty of Medicine, “Carol Davila” University of Medicine and Pharmacy, 050474 Bucharest, Romania; dana.balta@umfcd.ro (M.D.B.); ovidiu.bajenaru@umfcd.ro (O.B.); catalina.nuta@umfcd.ro (C.N.); octavian.andronic@umfcd.ro (O.A.); 3 Gastroenterology Department, University Emergency Hospital of Bucharest, 050098 Bucharest, Romania; 4 National Institute of Gerontology and Geriatrics “Ana Aslan”, 011241 Bucharest, Romania; 5 Gastroenterology Department, Clinical Emergency Hospital, 014461 Bucharest, Romania; christopher.pavel@gmail.com (C.P.); vasile.sandru@umfcd.ro (V.S.); 6 Department of Medical Disciplines, Faculty of Medicine and Pharmacy, University of Oradea, 410073 Oradea, Romania; mihaimunteanual@yahoo.com; 7 Center of Innovation and e-Health, Carol Davila University of Medicine and Pharmacy, 020021 Bucharest, Romania

**Keywords:** vitamin D, vitamin D receptor, pancreatic ductal adenocarcinoma, tumor microenvironment, epithelial-to-mesenchymal transition, stromal remodeling, cancer associated fibroblasts

## Abstract

Introduction: From the observation of a negative relationship between UV-B exposure and cancer rates, we hypothesized that vitamin D (VD) may play a protective role in oncogenesis. Moreover, repurposing a well-known and relatively safe drug for conditions with dismal prospects, such as pancreatic ductal adenocarcinoma (PDAC), is a tempting idea. Thus, we aimed to summarize the current knowledge regarding the role of VD in the prevention and treatment of PDAC. Methods: We conducted a systematic review of VD and PDAC using Medline-indexed studies accessed through PubMed as the primary data source. This study aimed to identify articles focusing on the role of VD as a risk and prognostic factor for PDAC, mechanistic studies evaluating the effects of VD or vitamin D analogs (VDAs) in PDAC models, and clinical trials on VDAs in PDAC. After the screening, 97 studies were included in the final manuscript. Conclusion: Even though the results from epidemiologic studies were contradictory, basic research has demonstrated that VD can act on PDAC cells either directly, inhibiting proliferation, apoptosis, EMT, migration, invasion, and stemness, or indirectly, through stromal remodeling. A better understanding of the consequences of VD-induced tumor–stroma cross-talk alterations is needed to determine whether VD/VDAs can be used to our own advantage in the treatment of PDAC.

## 1. Introduction

Known mainly as a key mediator of calcium homeostasis, vitamin D (VD) is responsible for a myriad of non-classical, non-hypercalcemic roles, ranging from immune system modulation to the regulation of cell proliferation and differentiation regulation [1,2]. These findings, supported by epidemiological studies showing a contrary relationship between UV-B exposure and cancer rates [3,4], generated the hypothesis that VD may possess anticarcinogenic effects.

First, we focused on colon cancer. As the results were promising, research was extended to other neoplasms as well. One such endeavor regards pancreatic cancer (PC), the 3rd deadliest malignancy in the US, with an overall survival rate at 5 years of 13% [5]. The dim prognosis is partly explained by its late diagnosis; 80% of cases are classified as locally advanced or disseminated from the first presentation. This restricts the availability of appropriate therapeutic options.

Thus, repurposing a well-known and relatively safe drug for conditions with poor prospects is a tempting idea. Thus, we aimed to summarize the present knowledge regarding VD and its role in the prevention and treatment of pancreatic ductal adenocarcinoma (PDAC), the most common type of PC, representing 92% of cases diagnosed in the US [5].

## 2. Material and Methods

To assess whether VD or VDAs may be considered possible candidates for PDAC treatment, we conducted a comprehensive literature review on their association. Our aim was to include articles reporting the following: 1. epidemiological studies linking VD status to the risk of developing PDAC, 2. epidemiological studies linking VD status to the risk of disease progression and outcomes 3. preclinical studies investigating the effects of VD or VDAs on in vitro and in vivo models of PDAC; 4.clinical trials of VDAs for PDAC.

**Figure 1:**
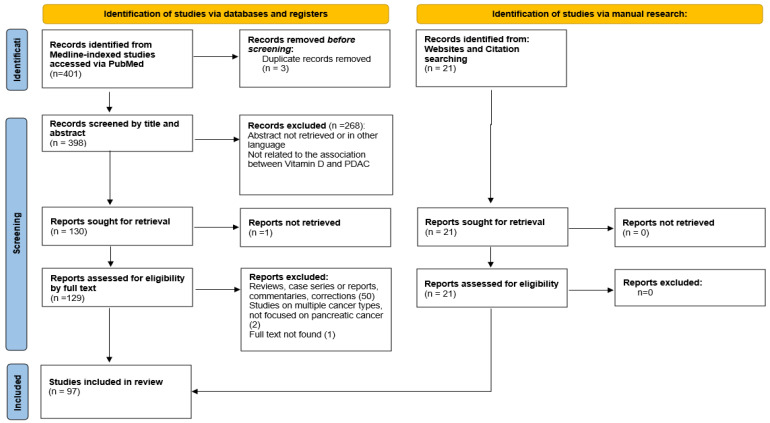
Flow-chart depicting the search strategy employed for article selection.

Our primary data source was Medline-indexed studies accessed through PubMed. Our search included terms were “vitamin D”, “25-hydroxyvitamin D”, “calcitriol”, “pancreatic ductal adenocarcinoma”, “pancreatic cancer”, “pancreatic neoplasms”, or “pancreatic malignancies”. Eligible studies were selected from the earliest available date to May 1, 2024, after title and abstract screening. Relevant articles identified through manual research of the reference lists were also included. After retrieval, the full text of the studies was reassessed. After evaluating and selecting articles based on form and content, we included 97 articles in the final manuscript (Figure 1).

## 3. Results and Discussion

### 3.1. VD Physiology

In humans, VD is predominantly produced through cutaneous synthesis and to a lesser degree via dietary intake. Endogenous production comprises the UV-B-light-mediated conversion of 7-dehydrocholesterol (7-DHC, provitamin D_3_) to vitamin D_3_ (cholecalciferol) in the skin [6] and is influenced by the UV-B exposure time, intensity, and skin pigmentation. Thus, factors, such as environmental conditions, latitude, time spent indoors, clothing, and sunscreen use, limit VD levels [7]. Exogenous sources of VD can be found in plants, mushrooms, and fortified foods, which contain vitamin D_2_ (ergocalciferol); fatty fish, containing only D_3_; and supplements, which may contain either D_2_ or D_3_. Dietary sources are important and must be noted as pharmacokinetics may differ. In addition, equivalent amounts of D2 have a lesser impact on serum VD levels than those of D3 [8].

To become biologically active, both D_2_ and D_3_ are converted to 1.5(OH)_2_-VD via a two-step hydroxylation process. First, hydroxylation at C25 occurs under the action of hepatic 25(OH)-hydroxylase (CYP2R1), resulting in the circulating form of VD (25(OH)-VD). A second hydroxylation at C1 occurs in the proximal convoluted tubule (PCT) under the action of 1(OH)-hydroxylase (CYP27B1) [9]. This step is regulated by FGF23, PTH, and feedback mechanisms. High 1,25(OH)_2_-VD levels inhibit CYP27B1 expression and simultaneously stimulate CY24A1 induction (24(OH)-hydroxylase responsible for VD inactivation) [10].

More recently, 1(OH)-hydroxylation has been observed in numerous extrarenal cells (pancreatic beta cells and ductal cells, osteoblasts, parathyroid cells, keratocytes, and macrophages, etc.) [11]. At this level, active VD usually acts in an autocrine or paracrine manner, as many CYP27B1-positive cells also express VD receptors (VDRs) [12]. Interestingly, extrarenal CYP27B1 is unresponsive to the same regulatory system as its renal counterpart, and active VD synthesis is dependent only on the substrate concentration [13,14]. Therefore, adequate VD levels are necessary to ensure normal functioning at these sites.

In circulation, 25(OH)-VD and 1,25(OH)_2_-VD are bound to VD Binding Protein (VBP) (in proportions of 88% and 85%, respectively) and albumin (12% and 15%, respectively). Very little serum VD is found in the free state (0.03% and 0.4%, respectively) [15]. Being fat-soluble, VD is generally taken up by cells via diffusion in its free state. A notable exception is PCT. VD bound to VBP is filtered by the glomerulus and then reabsorbed in the PCT by the megalin/cubilin complex [16].

As a sterolic hormone, most the actions of VD involve the modulation of gene expression. VD binds to its intracytoplasmic receptor (VDR), a recognized transcription factor (TF). Both D_2_ and D_3_ show similar affinities for VDR, complex partners with Retinoid X Receptor (RXR). The resulting heterodimer translocates into the nucleus and modulates gene transcription by binding to select regions of the genome—vitamin D response elements (VDREs) [17]. Non-genomic actions have also been described and are mediated by interactions with two receptors: VDR itself and membrane-associated rapid response steroid (MARRS)-binding protein [18,19].

Finally, all of these processes translate into classical, hypercalcemic, effects and in non-classical actions, such as the regulation of cell proliferation and differentiation or immune system modulation [1,2].

### 3.2. The VD Cancer Hypothesis

The hypothesis that vitamin D may be involved as a protective agent in cancer pathophysiology was first suggested in 1937, when Peller and Stephenson observed that US Navy personnel diagnosed with skin cancers (which they considered a substitute for extensive sun exposure) had decreased rates of other malignancies [3]. This idea was resurfaced in 1941, when Apperly et al. found a correlation between higher latitudes of residence and cancer mortality rates [4]. However, 40 years had passed since Garland et al. hypothesized that VD may be the missing link between solar radiation and cancer prevention [20]. The first studies to show such an association focused on colon cancers [20], but others soon followed, demonstrating a negative relationship between sunlight exposure (as a surrogate for VD level) and the risk of developing breast, ovarian, or prostate neoplasms [21,22,23]. These findings have attracted great interest regarding the anticarcinogenic properties of VD, and studies evaluating these effects have been extended to other malignancies, including PDAC.

### 3.3. Epidemiologic Studies on VD Levels and Risk of PDAC

Researchers have used multiple approaches to link VD status with PDAC incidence, ranging from nonspecific measures for estimating endogenous production and the exogenous intake of VD (UV-B exposure as a surrogate for the cutaneous synthesis and self-reported consumption of dietary sources of VD) to more encompassing evaluation tools, such as VD prediction scores and direct serum VD levels (see Table 1). In addition to environmental contributors, genetic variations in VD pathways have also been investigated as possible markers of the PDAC risk. Each method has its advantages and disadvantages, with studies yielding conflicting results.

Ecological studies based on published registries for UV-B radiation and cancer incidence represent an accessible method for assessing the role of VD as a risk factor for PDAC. UV-B radiation in the studies was estimated using astronomical, geographical, physical, and meteorological variables, such as the date of the equinox, geographical area of interest, average cloud cover, and estimates for solar radiation. Two large-scale studies (one from the US and Canadian databases and one from the IARC Global Cancer registries, including data from 175 countries) found an inverse association between the PC mortality rate and UV-B exposure [24,25]. However, a nested case-control study, which used the incidence of basal cell carcinoma (BCC) as a proxy for extensive sun exposure, noted no significant difference in PDAC incidence between patients with BCC and controls [26]. Despite the advantages of UV-B as a marker for VD (the major source of VD in humans, pre-existing databases), many variables interfere with the cutaneous synthesis of VD. Some factors may be accounted for (for example, phenotypic characteristics of the skin, age, seasonal variation, and adiposity), and others are more difficult to quantify (sun protection methods, time spent indoors, clothing style, etc., the conservative clothing of certain communities) and may cause confounding effects.

Another approach is based on estimating daily VD intake using self-reported questionnaires of daily nutrient intake in micrograms or the approximation of VD, intake calculated in international units from food supplements. One cohort study observed a lower PDAC risk with a higher VD intake [27], while two studies, including a large-scale pooled analysis, noted an increased PDAC risk with diets rich in VD [28,29]. Multiple other studies, including a large meta-analysis, have shown no association [30,31,32,33,34]. However, the method is limited by self-reporting bias (especially in retrospective studies, where the known cancer diagnosis may alter the patient’s perception of past habits) and by the oversight of endogenous VD production.

**Table 1:** Observational studies on vitamin D’s effect on the risk of pancreatic cancer (from Pubmed indexed articles, accessed on 1 May 2024).

Ref.	Study Design	Population	Participants	Results
UV-B exposure				
[24]	Ecologic	Men and women (US) North American Association of Central Cancer Registries 1998–2002		Weak evidence of increased PDAC incidence rates with lower UV-B exposure (RR = 1.09, 95% CI 1.05–1.14 in men and RR = 1.17, 95% CI 1.13–1.22, in women).
[26]	Nested case-control	Men and women (Sweden) National Swedish Cancer Registry 2004–2008	42 PDACs among 115,016 patiens with BCC— a proxy for extensive sun exposure. 241 PDACs among 987,893 controls.	No significant difference in PDAC incidence between BCC pacients and controls.
[25]	Ecologic	Men and women 175 countries IARC Global Cancer database		UV-B was independently inversely associated with incidence in men (*p* = 0.01) and women (*p* = 0.02).
VD intake				
[31]	Case-control	Men and women from The South Australian Cancer Registry 1984–1987	104 PDAC cases. 253 controls.	No association between VD intake and PDAC risk (compared to the 1st quartile, RR = 1.51 95% CI 0.76–2.99 for the 2nd, RR = 0.9 95% CI 0.43–1.86 for the 3rd, RR 1.49 95% CI 0.75–2.96 for the 4th, *p* = 0.51).
[27]	Cohort	Men 40–75 years (US) Health Professionals Follow-up Study (HPFS) 1986–2000 Women ages 38–68 years (US) Nurses’ Health Study (NHS) 1984–2000	365 PDACs (178 women in the NHS cohort, 187 men in the NPFS cohort).	Lower PDAC risk with higher VD intake (compared with total intake <150 IU/d, RR 0.78 (95% CI 0.59–1.01) for 150–299 IU/d, 0.57 (95% CI, 0.40–0.83) for 300–449 IU/d, 0.56 (95% CI, 0.36–0.87) for 450–599 IU/d and 0.59 (95% CI, 0.40–0.88) for >600 IU/d (*p*-trend = 0.01)).
[30]	Nested case-control	Male smokers 50–69 years (Finland) Alpha-Tocopherol, Beta-Carotene Cancer Prevention Study (ATBC) 1985–1997	167 PDACs.	No association between dietary VD intake and PDAC.
[28]	Case-control	Men and women 21–85 years (San Francisco Bay Area) 1995–1999	532 PDACs 1701 controls matched for age, Sex, and county controls.	In men, PDAC risk increased with dietary VD (highest (>450 IU/d) vs. lowest (<150 IU/d) OR = 2.6, *p*-trend = 0.009). For total VD (food and supplements), risk increased until the highest category of intake (>800 IU/d), where risk of PDAC was similar to the lowest category (<200 IU/d). No statistically significant associations were observed among women.
[32]	Case-control	Men and women from Northern Italy 1991–2008	326 PDAC cases. 652 controls matched for sex, age, and study center.	No association between dietary/total VD intake and PC risk was observed (compared to the 1st quartile, OR = 1.22 (0.73–2.03) for the 2nd, OR = 1.63 (1.00–2.67) for the 3rd, OR = 1.51 (0.92–2.49) for the 4th, OR = 1.34 (0.82–2.20) for the 5th, *p* trend = 0.19, OR continuous 1.03 (0.89–1.19)).
[33]	Case-control	Men and women from Mayo Clinic, USA 2004–2009	384 PDAC cases. 983 controls matched for age, race, sex, and residence.	No association between dietary/total VD intake and PDAC risk was observed (compared to the 1st quartile, OR = 1.15 (0.78,1.69) for the 2nd, OR = 0.95 (0.64,1.42) for the 3rd, OR = 0.95 (0.64,1.41) for the 4th, OR = 1.04 (0.70,1.54) for the 5th, *p*-trend = 0.81).
[34]	Pooled analysis	14 cohort studies	2212 PDAC cases, among 862 680 individuals.	No association between dietary/total VD intake and PDAC risk was observed.
[29]	Pooled analysis	Men and women from 9 case-control studies—International Pancreatic Cancer Case-Control Consortium- 6 studies from US, 1 Italy, 1 Australia, 1 multi-national.	2963 PDACs 8527 controls.	PDAC risk increased with VD intake (per 100 IU/day: OR = 1.13, 95% CI 1.07–1.19, *p* < 0.001; ≥230 vs. <110 IU/d: OR = 1.31, 95% CI 1.10–1.55, *p* < 0.001). No association between PC risk and supplementary vitamin D intake (4 studies).
[35]	Meta-analysis	[15,29,34,36,37,38,39]	17,385 PDAC patiens	Dietary VD had no correlation with PDAC risk (RR = 1.11, 95% CI = 0.67–1.86).
[40]	Meta-analysis	3 prospective studies [27,41,42], 6 retrospective studies [28,31,32,33,38]		In prospective studies, RR for VD levels and PDAC risk was 0.74 (95% CI: 0.52–1.06). However, in retrospective ones, RR was 1.21 (95% CI: 0.97–1.51).
Serum VD levels				
[41]	Nested case-control	Men, smokers, 50–69 years (Finland) ATBC Study 1985–2001	200 PDACs. Two controls matched by age and month of blood draw.	Higher VD levels presented with a 3-fold increased PDAC risk (highest vs. lowest quintile, >65.5 vs. <32.0 nmol/L: OR, 2.92; 95% CI, 1.56–5.48, *p*-trend = 0.001).
[38]	Nested case-control	Men and women 55–74 years (US) Prostate, Lung, Colorectal, and Ovarian Screening Trial (PLCO) 1994–2006	184 PDAC cases. Two controls matched by age, race, sex, and date of blood draw.	No association was found between VD levels and PDAC incidence (highest vs. lowest quintile, >82.3 versus <45.9 nmol/L: OR, 1.45; 95% CI 0.66–3.15; *p*-trend = 0.49).
[43]	Pooled nested case-control	Men and women from 8 cohorts within the Cohort Consortium Vitamin D Pooling Project of Rarer Cancers (VDPP) 1974–2006	952 PCs 1333 controls.	High VD concentration (>100 nmol/L) presented 2-fold increase in PC risk overall (OR 2.12, 95% CI: 1.23–3.64).
[39]	Pooled nested case-control	Men and women from 5 cohorts through 2008	451 PDACs 1167 controls.	Compared with those with VD levels < 50 nmol/L, ORs were 0.75 (0.58–0.98) for subjects with 50–75 nmol/L levels and 0.71 (0.52–0.97) for those with levels > 75 nmol/L. No increased risk was noted in subjects with >100 nmol/L.
[44]	Nested case-control	Men, smokers, 50–69 years (Finland) ATBC Study 1985–2005	123 PDACs. 123 controls matched by age and month of baseline blood draw.	PDAC risk increased with VD levels, although none of the patterns appear monotonic, and statistical significance was achieved for the highest quartile only (OR for the highest vs. lowest quintile was 2.01, 95% CI 1.08–3.72, *p*-trend = 0.03).
[15]	Nested case-control	Men and women 55–74 years (US) PLCO Study 1994–2010	295 PDACs. Two controls matched by age, race, sex, and month of blood draw.	Compared with the group with VD levels between 50 and 75 nmol/L, the highest group had a significantly higher risk (>100 nmol/L; OR: 3.23; 95% CI: 1.24, 8.44), whereas in the lowest group, no significant risk was noted (<25 nmol/L; OR: 2.50; 95% CI: 0.92, 6.81).
[37]	Nested Case-control	Men and women 20–100 years The Copenhagen City Heart Study 1981–1983 With follow-up 1991–1994, 2001–2003	109 PDACs. 9682 controls.	Multivariable adjusted PDAC HRs calculated for a 50% reduction in VD concentration were non-significant (HR 1.05, 95% CI 0.84–1.3, *p* > 0.05).
[45]	Meta-analysis	[26,27,28,38,39,41,42,43,44]		The pooled OR of PDAC for the highest vs. the lowest categories of VD level was 1.14 (95% CI 0.896–1.451); the Z-score for the overall effect was 1.06 (*p* = 0.288), showing no significant association between VD levels and PDAC risk.
[36]	Cohort	Men and women with IBD Great Boston Area 1986–2014	13 PDACs. 2796 controls.	No significant increase in PDAC risk was noted for each 1 ng/mL increase in plasma VD levels (HR 0.97, 95% CI 0.92–1.02, *p* = 0.26).
[35]	Meta-analysis	[15,29,34,36,37,38,39]	17,385 PDAC patients.	VD levels (RR = 1.02, 95% CI = 0.66–1.57) did not correlate with PDAC risk (RR = 1.11, 95% CI = 0.67–1.86).
[46]	Pooled nested Case-control	Men and women from European Prospective Investigation into Cancer and Nutrition (EPIC) 1992–2009 and the Nord-Trøndelag Health Study’s second survey (HUNT2) cohorts 1995–2007	738 PDACs. 738 controls matched by by center, sex, follow-up, age, and fasting status at blood collection	Compared with the group with VD levels of 50–75 nmol/L, the adjusted OR (95% CIs) was 0.71 (0.42–1.20) for VD levels < 25 mmol/L, 0.94 (0.72–1.22) for 25–50 mmol/L, 1.12 (0.82–1.53) for 75–100 mmol/L, and 1.26 (0.79–2.01) for >100 nmol/L, respectively (*p*-trend = 0.09).
[47]	Case—control	Men and women from multiple medical centers in Egypt that presented for investigation of pancreatic lesions.	47 PDAC cases. 37 controls.	Low serum VD did not increase PDAC risk.
[48]	Cohort	Men and women 40–70 with metabolic syndrome from the UK Biobank Cohort 2006–2010	257 PDACs, among 97,621 participants.	No correlation between PDAC incidence and VD levels was found in patients with MetS.
[49]	Meta—analysis	[15,26,38,39,41,42,44,46,50]	10,587 PDAC cases, among 529,917 participants.	The pooled OR of PDAC for the highest vs. the lowest categories of VD serum levels was 0.98 (95% CI 0.69–1.27).
Estimated VD				
[50]	Cohort	Male dentists, optometrists, osteopaths, podiatrists, pharmacists, and veterinarians, 40–75 years HPF Study 1986–2000	4286 total cancers among 47 800 participants.	Lower VD scores increased PDAC risk (RR = 0.49, 95% CI = 0.28–0.86).
[42]	Cohort	Female registered nurses 30–55 years NHS Study 1986–2006 Male dentists, optometrists, osteopaths, podiatrists, pharmacists, and veterinarians, 40–75 years, HPF Study 1986–2006	575 PDACs among 118,597 participants.	PDAC risk decreased with higher VD scores (highest vs. lowest quintile RR of 0.65, 95% CI 0.50–0.86; *p*-trend 0.001).
[25]	Ecologic	Men and women from 175 countries IARC Global Cancer database		Incidence rates were half as high in countries with estimated serum VD >30 ng/mL than in those <30 ng/mL.
**VD genetic markers**				
[51]	GWAS	Men and women from 3 major hospitals in Shandong, China 2010–2012	91 PDACs. 80 controls matched for age and sex.	Patients with heterozygous variants of VDR SNPs FokI (rs2228570) had a decreased risk of PDAC. BsmI(rs1544410) SNPs were not associated with PDAC risk.
[52]	GWAS	Ontario Pancreas Cancer Study 2002–2009	628 PDACs. 1193 controls matched for age and sex.	SNPs in CYP24A1, CYP2R1, CASR, VDB, RXRA, and LRP2 genes were associated with PDAC risk. However, after multiple comparison adjustment, no significant association was noted.
[53]	GWAS	10 cohort studies and 10 case control studies in the PanScan collaboration.	3583 PDACs. 7053 controls	No examined gene involved in VD’s pathway was associated with PDAC risk. Individual SNPs (VDR—rs2239186, LRP2—rs4668123, CYP24A1—rs2762932, GC—rs2282679 and CUBN—rs1810205) were associated with PDAC incidences (*p* values 0.008–0.037), but after multiple comparison adjustments, no significant association was noted.
[54]	GWAS	Men and women from 2 major hospitals in Shandong, China 2006–2012	258 PDACs. 385 controls matched for age and sex.	In VDR SNP rs2228570, the T loci and the genotypes with T alleles increased the risk of PDAC, while in VDR SNP rs1544410, the G loci and genotypes AG + GG decreased the risk.
[55]	Meta-analysis	29 studies included on CYP24A1 and cancer risk, however only one on PDAC [52]		CYP24A1 SNP rs2585428 significantly decreased the risk of PDAC (see [52]).
[47]	GWAS	Men and women from multiple medical centers in Egypt that presented for investigation of pancreatic lesions.	47 PDACs. 37 controls.	VDR SNP Fok1 rs2228570 did not correlate with PDAC risk.
[56]	Mendelian randomisation	Men and women from Pancreatic Cancer Cohort Consortium Genome-Wide Association Study (PanScan)	1896 PDACs. 1939 controls.	OR per 25 nmol/L increase in genetically determined VD levels was 1.36 (95% CI 0.81–2.27, *p* = 0.25).
[57]	Mendelian randomization	Men and women 37–73 years The UK Biobank (UKB) 2006–2010	500 PDACs.	OR per 20 nmol/L increase in genetically determined VD levels was 1.09 (0.63–1.88, *p* = 0.76).
[58]	Mendelian randomisation	IEU OPEAN GWAS PROJECT Databank	605 PDACs. 218,187 controls.	No significant association between genetically determined VD levels and PDAC risk was noted (*p* = 0.905, OR = 0.969, 95% CI 0.581–1.618)

Studies based on serum VD levels have also shown contradictory results. While the vast majority of studies noted no statistically significant association [35,36,37,38,40,45,46,47,48,49,59], one pooled nested case-control study found an opposing relationship between VD serum values and PDAC risk [39], while four others showed an increased risk of PDAC with higher serum VD levels [15,41,43,44]. In the latter studies, PDAC patients were compared to controls matched for age, sex, time of serum measurement, and race to minimize the effect of UV-B exposure confounders. However, these results were not positive monotonic, with statistical significance achieved in most studies for the highest subgroup only [15,43,44]. In addition, some reports opted to divide the investigated population by means of “clinically relevant” VD intervals rather than centiles, generating groups of widely varying sizes (see Table 2). Notably, the higher-end VD level subgroup had the smallest sample size, raising the question of whether the observations are merely statistical artifacts. Another methodology limitation among studies is the variation among the techniques used to determine the serum VD level (Table 2). Most studies used immunoassay kits as they are widely available and cost affective, but they lack the accuracy and specificity of other more complex and expensive tests, such as liquid chromatography tandem mass spectrometry. Another important limitation of these epidemiological studies was the heterogeneity of the examined populations (e.g., Finnish male smokers, US-based female nurses, and male healthcare providers).

Not to be overlooked is the fact that all studies analyzed the association and not the causality between VD concentrations and PDAC incidence. The cause–effect relationship is difficult to assess in such studies, as there are many variables that may explain the low VD concentrations in this population. In most studies, treatment-related deficiency (such as malabsorption associated with surgery or impaired VD metabolism due to drug-induced hepato- or nephrotoxicity) was excluded by measuring VD levels at diagnosis. However, other factors, such as the degree of exocrine pancreatic dysfunction or reduced sun exposure due to an altered general status, cannot be accounted for. Moreover, serum levels of VD may not be an accurate measure of active VD levels (1,25(OH)_2_-VD), as genetic traits or pathology may influence conversion. In addition, a single measurement may not correlate with long-term average vitamin levels.

Studies based on VD serum level prediction scores seemed to yield more uniform results, with three studies showing an increased PDAC risk with lower estimations of VD levels [25,42,50]. These scores were established using linear regression models and included dietary and supplementary vitamin D, skin pigmentation, body mass index (BMI), geographic residence, and time spent outdoors (for sunlight exposure estimation). Theoretically, such models should correlate better with long-term average VD levels than with just one serum VD measurement.

However, these approaches fail to consider individual differences in VD’s metabolism and pathways of VD. As twin studies have estimated that genetics may be accountable for 20–85% of serum VD levels [60], genotype involvement in the interplay between VD and PDAC should also be examined. Multiple genome-wide association studies (GWASs) investigating single nucleotide polymorphisms (SNPs) of genes involved in the VD pathway, such as VDR, CYP24A1, CYP2R1, CASR (calcium sensing receptor encoding gene), VDB, RXR, and LRP2 (megalin encoding gene), noted no correlation with PDAC risk [47,52,53]. In contrast, two studies performed by the same research team observed that Fok1 (rs2228570) genotypes correlated with PDAC risk [51,54]. Fok1 is the only SNP of the VDR gene that is known to alter the protein structure. It is located at the initiation codon in two variants, ACG (*F*-VDR) or ATG (*f*-VDR). The *F*-VDR protein is three amino acids shorter and more active, whereas the longer version *f*-VDR is less responsive [61]. In both studies, patients with genotypes with T alleles (ff or Ff) had an increased risk of PDAC. Another important VDR SNP was Bsml (rs1544410). Located in intron 8; this SNP does not alter the protein structure of VDR but impacts its expression through mRNA instability [61]. In one study, Li et al. [54] observed that patients with genotypes with G alleles (bb or Bb) have a decreased risk of developing PDAC. In another study [51], no significant results were observed for Bsml alone. However, when investigating the cumulative effect of Fok1 and Bsml, a higher PDAC incidence was noted in the ffbb and Ffbb populations [51].

Despite conflicting data, some researchers went a step further and tried to prove causality between low VD levels and PDAC incidence using Mendelian randomization (MR) studies [56,57,58]. This approach eliminates the impact of confounders and reverse causalities, which are difficult to quantify in traditional observational studies (e.g., medical conditions restrain activity, decrease sun exposure, and lead to low VD levels). Scores based on SNPs known to influence serum VD levels were defined. Assuming random gene assortment at conception, and cases and controls were divided by these scores and compared depending on the outcome (PDAC diagnosis). No association between genetically determined VD levels and PDAC risk was found in three [56,57,58].

### 3.4. Epidemiologic Studies on VD Levels and Outcome of PDAC

VD status was examined not only as a risk factor for PDAC but also as a predictive factor. Unfortunately, both investigations have yielded conflicting results (see Table 3).

Multiple ecological studies assessing sun exposure in certain populations (either by latitude or UV-B radiation) noted an inverse correlation with PDAC mortality [24,62,63,64,65,66,67], while one based on registries from Australia observed higher PDAC mortality rates with UV-B exposure [68].

Studies based on serum VD levels have also shown contradictory results. While some studies found no significant association [59,69,70,71,72], others noted that patients with higher VD levels had better outcomes [35,49,73,74,75,76,77,78]. However, these results are highly heterogeneous: cut-offs for dividing the population based on plasma VD levels, the technique used for the VD measurement (see Table 4), the PDAC population investigated (early or advanced cases, with/without chemoradiotherapy [CRT]), and clinical end points used (overall survival [OS], progression-free survival [PFS], time to progression [TTP], and distant metastasis-free survival [DMFS]) vary among studies.

An important limitation of the aforementioned approaches is their inability to account for all confounders. Thus, any association noted, although statistically significant, may be a spurious observation. To avoid bias caused by confounding factors, studies with different designs were conducted. For example, some research teams have assessed the role of the tumor expression of proteins involved in the VD pathway in PDAC prognosis. Three studies evaluated the role of VDR [79,80,81]: two small-sample studies noted improved OS with higher VDR expression [79,80], while one large study involving PDAC patients from The Cancer Genome Atlas observed the opposite effect [81]. Bahat et al. examined VDR expression in desmoplastic tissue surrounding cancerous cells and found a positive association with OS only in N0 disease cases [79,80]. CYP24A1, the enzyme that inactivates active VD, is another protein for which the role in PDAC outcomes has been investigated. Gao et al. found that CYP24A1 expression correlated with OS in a multivariate analysis [82].

Another approach used to minimize bias targeted the SNPs of genes involved in the VD pathway and their involvement in PDAC prognosis. In our review of the literature, three such studies were found: two of them noted no association between the investigated SNPs and PDAC outcome [47,74], but the third one observed that OS correlated with the VDR variant rs2853564. More precisely, patients with the GG genotype had a better OS than those with the AA genotype [83]. In the present study, the G allele of this SNP showed increased transcriptional activity of the VDR gene. In addition, the VDR variant with the G allele showed a decreased affinity for the interferon regulatory factor 4 (IRF4) transcription factor [83].

**Table 3:** Observational studies on vitamin D’s effect on the outcome of pancreatic cancer (from Pubmed indexed articles, accessed on 1 May, 2024).

Ref.	Study Design	Population	Participants	Results	
UV-B exposure					
[62]	Ecologic study	Men and women (US)—Atlas of Cancer Mortality in the United States 1970–1994		PDAC premature mortality rates increased with lower UV-B exposures and in African-Americans (r = −0.33, *p* < 0.001).	
[63]	Ecologic study	Men and women (15 countries in Europe)—WHO Databank 1989–1991		Multiple linear regression models were established between PDAC mortality rates and latitude, fish, fruit, and alcohol consumption in men (r = 0.97, *p* < 0.001) and between PDAC mortality rates and latitude and fish consumption in women (r = 0.896, *p* < 0.001).	
[64]	Ecologic study	Men and women (15 countries in Europe)—WHO Databank 1989–1991		In multivariate linear regression models, UV-B exposure and fish consumption reduced PDAC mortality in men (adjusted r^2^ = 0.75, *p* < 0.001), while UV-B exposure decreased PDAC mortality in women (adjusted r^2^ = 0.71, *p* < 0.001)	
[65]	Ecologic study	Men and women (47 prefectures from Japan) 2000		Inverse correlation between UV-B exposure and mortality rates in men (r = −0.51, *p* < 0.01) and women (r = −0.32, *p* < 0.05).	
[24]	Ecologic study	Men and women (US)—National Cancer Institute’s Stat database 1993–2002		Weak evidence of an inverse association between PDAC mortality rate and UV-B exposure (RR = 1.06, 95% CI 1.03–1.09 in men and RR = 1.11, 95% CI 1.08–1.14, in women).	
[66]	Ecologic study	Men and women (Japan)—National Vital Statistics database 1998–2002		Regression coefficients for the amount of global solar radiation and the daily maximum temperature were −4.35 (*p* = 0.00034) and −2.81 (*p* < 0.0001), respectively, in males and −5.02 (*p* < 0.0001) and −1.87 (*p* < 0.0001), respectively, in women.	
[67]	Ecologic study	Men and women from 48 continental Spanish provinces 1978–1992		PDAC mortality rates correlate with latitude (r = 0.55, *p* < 0.01).	
[68]	Ecologic study	Men and women (Australia)—Australian General Record of Incidence and Mortality books 1968–2005		Significant association between vitamin D-effective UV radiation and PC mortality, with an estimated 1.5% decrease in the risk per 10-kJ/m^2^ increase in yearly vitamin D-effective UV radiation.	
Serum VD					
[73]	Case-control	PDAC patients—Siteman Cancer Center in St. Louis, Missouri 2007–2011	174 PDAC cases	VD levels < 20 ng/mL were associated with a poorer prognosis (*p* = 0.0019) in PDAC stage III and IV.	
[72]	Nested case-control	PDAC patients—CALGB 80303	256 PDAC cases	VD levels were not associated with PFS (HR = 1.00, 95% CI = 0.99–1.01, *p* = 0.60) or OS (HR = 1.00, 95% CI = 0.99 to 1.01, *p* = 0.95).	
[69]	Case-control	Advanced PDAC patients—Center, Ludwig-Maximilians-University of Munich 2009–2012	59 advanced PDAC cases	VD did not influence OS in PDAC.	
[70]	Case-control	PDAC patients—Cancer Treatment Centers of America hospitals 2011–2014	627 PDAC cases without prior cancer-directed therapy	VD levels did not influence OS or TTP in PDAC.	
[74]	Pooled nested case-control	PDAC patients from five US prospective cohort studies (HPFS, NHS, PHS, WHI, and WHS)	493 PDAC cases	Mortality HRs were 0.79 (95% CI, 0.48–1.29) for VD levels between 20 and 30 ng/mL and 0.66 (95% CI, 0.49–0.90) for levels over 30 ng/mL, when compared to insufficient VD levels (below 20 ng/mL) (*p_trend_* = 0.01).	
[35]	Meta-analysis	[69,70,72,73,74]	1613 PDAC cases	The high VD level group had reduced PDAC mortality compared to the reduced level group (HR = 0.81, 95% CI = 0.68–0.96, I^2^ = 43.0%).	
[78]	Case-control	PDAC patients with/without CRT who underwent curative surgery Osaka, Japan, 2007–2012	86 PDAC cases	In patients who received CRT, higher preoperative plasma VD levels correlated with DMFS.	
[75]	Nested case-control	PDAC patients from BIOPAC database in Denmark 2008–2018	1271 PDAC cases	In stages I and II, patients with VD deficiency (<25 nmol/L) had reduced OS compared to those with sufficient status (>50 nmol/L) (18.3 vs. 29.7 months, HR = 1.55, 95% CI 1.04–2.31; *p* = 0.03).	
[59]	Nested case-control	Male smokers 50–69 years (Finland) Alpha-Tocopherol, Beta-Carotene Cancer Prevention Study (ATBC) 1985–1993	143 PDACs	Survival increased with higher levels of VD, but the results were not statistically significant.	
[71]	Post hoc analysis	PDAC patients from phase III MPACT trial of metastatic pancreatic cancer	422 PDAC cases	No difference in OS was observed with relatively insufficient (20–30 ng/mL) or sufficient (>30 ng/mL) versus insufficient 25(OH)D levels (*p* = 0.227, respectively 0.74) or with sufficient vs. relatively insufficient (*p* = 0.301).	
[76]	Case-control	PDAC patients from El-Mabra Hospital, Egypt 2016–2018	176 advanced PDAC cases	Lower VD levels were associated with poor outcome: compared to VD > 30 ng/mL, HR = 1.974 (95% CI: 1.362–2.862, <0.001) for VD levels 20–30 ng/mL, respectively, 2.661 (95% CI: 1.362– 2.862, <0.001) for VD levels <20 ng/mL.	
[77]	Meta-analysis	[71,72,73,74,75,76]	2369 advanced PDAC cases	VD levels positively correlated with OS (HR 2.37; 95% CI 2.22–2.54; *p* < 0.001). VD levels did not impact PFS.	
[49]	Meta-analysis	[59,69,70,71,74,75]		The pooled OR of PC for the highest (>65 nmol/L) vs. the lowest categories (<35 nmol/L) of VD level was 0.64 (95% CI 0.45–0.82) in cohort studies and 0.78 (95% CI 0.62–0.95) in case-controls.	
VD metabolic pathway components’ expression in PDAC tissues					
[79]	Case-control	PDAC patients from the First Hospital of China Medical University 2006–2012	Tissues samples from 61 PDAC patients	Significantly longer OS was observed in cases with high tumor VDR expression compared to those with low or no VDR expression (*p* = 0.037).	
[80]	Case-control	PDAC patients operated between at the Tel Aviv Sourasky Medical Center confirmed with desmoplasia 2009–2017	Tissues samples from 127 PDAC patients	High VDR expression was associated with improved OS (median 33; 95% CI = 26.4–39.6 vs. 18; 15.5–20.5 months, *p* = 0.01) and DFS (median: 22; 95% CI = 13.7–30.3 vs. 8; 95% CI = 5–11 months, respectively, *p* = 0.015) in localized disease. No significant effect was noted when the whole population was examined.	
[81]	Nested case-control	PDAC patients from The Cancer Genome Atlas		VDR expression was associated with poorer OS (*p* = 0.035) and DFI (*p* = 0.033).	
[82]	Case-control	PDAC patients with no previous CRT operated at the General Hospital of PLA, CHINA	Tissues samples from 73 PDAC patients	Percentage of CYP24A1 -ositive cells (*p* = 0.023) and average staining intensity of the CYP24A1-positive cells (*p* = 0.026) in PDAC tissues significantly correlated with survival. However, in univariate survival analysis, no significant association was noted.	
Polymorphisms of genes involved in VD’s pathway					
[74]	Pooled nested case-control	PDAC patients from five US prospective cohort studies (HPFS, NHS, PHS, WHI, and WHS)	493 PDAC cases	No significant association between VDR SNPs and survival was found (for corrected significance threshold of *p* = 0.002).	
[83]	Case-control	PDAC patients from from the Cancer and Leukemia Group B (CALGB) 80303 * and the Mayo Clinic **	294 PDAC cases * 408 PDAC cases **	VDR SNP rs2853564 was associatedwith OS in PDAC patients (HR 0.74, 0.63 ± 0.87, *p* = 0.0002 * and HR 0.81, 95% CI 0.70 ± 0.94, *p* = 0.0059 **).	*/** Increased survival with higher levels of VD, but not statistically significant.
[47]	Case-control	Men and women from a multiple Medical centers in Egypt that presented for investigation of pancreatic lesions.	47 PDAC. 37 controls.	VDR SNP Fok1 rs2228570 din not correlate with PDAC survival.	

**Table 4:** Methodology differences in studies assessing VD levels and PDAC outcome.

Ref.	Technique for Quantitative Determination of VD Levels	Approach Used for Dividing the Population by Means of VD Level
[73]	No mention	Intervals set as <20 ng/mL (50 nmol/L), 20–29.9 ng/mL (50–75 nmol/L), ≥30 ng/mL (75 nmol/L)
[72]	Direct Competitive Chemiluminescent Immunoassay	Intervals set as <20 ng/mL (50 nmol/L), 20–29.9 ng/mL (50–75 nmol/L), ≥30 ng/mL (75 nmol/L)
[69]	Electrochemiluminescence binding assay	Used a lower limit of normal of 10 ng/mL
[70]	No mention	Intervals set as < 20 ng/mL, 20–40 ng/mL, 40–60 ng/mL, ≥60 ng/mL
[74]	Enzymeimmunoassay	Intervals set as <20 ng/mL (50 nmol/L), 20–29.9 ng/mL (50–75 nmol/L), ≥30 ng/mL (75 nmol/L)
[35]	No mention	Quartiles
[78]	No mention	VD levels used as a continuous variable
[75]	No mention	Intervals set as < 25 nmol/L, 25–50 nmol/L, ≥ 50.0 nmol/L
[59]	Direct Competitive Chemiluminescent Immunoassay	Quartiles
[71]	Liquid chromatography-tandem mass spectrometry	Intervals set as <20 ng/mL (50 nmol/L), 20–29.9 ng/mL (50–75 nmol/L), ≥30 ng/mL (75 nmol/L)
[76]	Enzyme immunoassay	Intervals set as <20 ng/mL (50 nmol/L), 20–29.9 ng/mL (50–75 nmol/L), ≥30 ng/mL (75 nmol/L)
[77]	Liquidchromatography-tandem mass spectrometry Enzyme immunoassay Direct Competitive Chemiluminescent Immunoassay	Intervals set as <20 ng/mL (50 nmol/L), 20–29.9 ng/mL (50–75 nmol/L), ≥30 ng/mL (75 nmol/L)
[49]	No mention	Intervals set as highest category: >65 nmol/L lowest category: <35 nmol/L

### 3.5. A Model of PDAC Progression and the Molecular Mechanisms of Vitamin D’s Anticarcinogenic Effect in Pancreatic Cancer

PDAC tumorigenesis typically follows a model of progression from precancerous lesions to invasive disease through a complex process of accumulating genetic and epigenetic alterations over time.

The first step is acinar-to-ductal metaplasia (ADM), which involves the transdifferentiation of acinar/centroacinar cells into duct-like cells. Subsequently, as cellular atypia accumulates, ADM transforms into pancreatic intraepithelial neoplasia (PanIN) [84].

The genetic profile of PDAC is highly variable. Tumorigenesis is generally initiated by activating KRAS mutations (90% of PDAC cases), favoring PanIN formation. The gain of KRAS function impairs GTP-ase activity and upregulates downstream pathways (e.g., MAP, PI3K/AKT/mTOR) involved in cell transformation, proliferation, and metastasis [85,86]. Progression from low-grade PanIN to high-grade PanIN and finally to PDAC is mediated by various inactivating mutations in tumor suppressor genes, such as TP53, CDKN2A, and SMAD4 [85]. Mutated TP53 cells lose the ability to upregulate p21 synthesis, a cyclin-dependent kinase (CDK) inhibitor, in response to DNA damage. Thus, without functional proteins, malignant cells elude cell cycle arrest and apoptosis. CDKN2A mutations also result in the dysregulation of another CKD inhibitor (2A), responsible for blocking cell entry into S-phase division [85,86]. SMAD4 is a central mediator of the TGF-β signaling pathway and participates in the regulation of cell cycle arrest, apoptosis, and the cytotoxic immune response [86].

PDAC cells also undergo epithelial-to-mesenchymal transition (EMT). EMT is a crucial process characterized by phenotypic changes from epithelial cells to mesenchymal cells. By downregulating adhesion molecules, such as E-cadherin, and increasing mesenchymal markers, such as N-cadherin and vimentin, cells lose their polarity and ability to adhere to one another. Thus, delamination from ductal structures into the adjacent stroma is possible, leading to invasive carcinoma [84]. The signaling pathways involved in EMT are Wnt/β-catenin, which is mediated by FoxM1 (Wnt3a increases FoxM1, which binds directly to β-catenin and enhances β-catenin nuclear localization and transcriptional activity) [87] and Hedgehog signaling (Shh/Ptch1/Smo). They also promote an enhanced stem-like phenotype in PDAC cells (stemness) [84,88]. At least three families of TFs have been recognized as triggers: Snail/Slug, ZEB1/2, and Twist families [89].

Simultaneously with tumorigenesis, a desmoplastic reaction occurs around the lesion, leading to the formation of a tumor fibroinflammatory microenvironment (TME) comprising cancer-associated fibroblasts (CAFs), endothelial and immune cells, and the extracellular matrix (ECM). This stroma accounts for 50–80% of the tumor volume and prevents the infiltration of chemotherapy into the lesion [90,91].

Initially, CAFs were believed to be activated pancreatic stellate cells (PSCs), which, once stimulated, lose their vitamin A storage capacity and start expressing α-smooth Muscle Actin (α-SMA), a marker used for CAF identification. However, recent studies have shown that only a small proportion of CAFs is derived from PSCs. Resident tissue fibroblasts, mesenchymal stem cells (MSCs), bone marrow-derived macrophages, adipocytes, endothelial cells, and pericytes can also differentiate into CAFs, which represent important sources [92].

Cross-talk exists between malignant cells and the TME. PDAC tumor cells recruit, by means of paracrine signaling, mesenchymal cells to differentiate into CAFs. Conversely, activated CAFs benefit tumor growth by ensuring metabolic support, promoting proliferation, and triggering EMT in tumor cells [90,92]. Moreover, CAFs deregulate the local immune system, recruiting immunosuppressive cells, such as myeloid-derived tumor suppressor cells (MDSCs), tumor-associated macrophages (TAMs), or T regulatory lymphocytes, and prevent the penetration of dense stromal cytotoxic T lymphocytes [92]. CAFs also exhibit tumor-limiting effects. The CAF-induced expression of collagen type I in the stroma physically restrains the growth and spread of malignant cells [93]. The anti-tumor effects of CAFs were proven in vivo when stromal depletion led to increased tumor aggressiveness [94].

When investigating the role of VD in PDAC, conflicting results regarding VD’s association between VD and PDAC risk and outcomes presents a dilemma: is there truly no connection between VD and PDAC, with the statistically significant results being mere coincidences, or are VD effects actually more complex and context-dependent, making it inaccurate to evaluate them using large, heterogeneous samples? Mechanistic studies were conducted for further clarification.

First, PDAC cells have been proven to possess molecular structures involved in the pathway of action of VD, such as VDR and 1(OH)-hydroxylase [95,96,97]. Having established that VD has the potential to act on PDAC cells, studies have focused on assessing its actual effects.

Multiple in vitro studies on human PDAC cell lines and in vivo studies using mouse xenograft models have demonstrated VD’s antiproliferative effect of VD in PDAC (see Table 5). A possible explanation for this effect is G0/G1 cell cycle arrest via upregulation of the CDK inhibitors p21 and p27 [96,98,99,100,101,102]. This mechanism is independent of the p53 level [95]. In addition, KRAS mutations do not offer any advantage [96]. Other possible mechanisms involved are Hedgehog signaling inactivation (by binding to and inhibiting Smo, independent of VDR) [103], Akt/PI3K pathway inhibition [79,104,105], Wnt/β-catenin pathway downregulation [79,106] (Arensman et al. suggest that this effect is caused by LRP6 expression reduction [106]) or FoxM1 signaling suppression [107]. He et al. found that VDR is silenced at the transcriptional level in VD-resistant PDAC cell lines, possibly due to epigenetic modifications (histone H3K27 hypoacetylation or DNA methylation). Reversing these processes increases the antiproliferative effect of VD on PDAC cells [108].

The effect of VD on malignant cell apoptosis was also investigated, but with mixed results: Chiang et al. and Pettersson et al. did not observe any apoptosis induction [101,109], Wang et al. found an apoptotic effect mediated by Bax/Bcl-2 pathway deregulation [79], Yu et al. noted that VD enhanced gemcitabine-induced caspase-dependent apoptosis [104], and Mouratiditis et al. noticed that VD counteracts retinoic acid-induced apoptosis [110].

Two studies also demonstrated the ability of VD to inhibit PDAC tumor cell migration and invasion [89,107]. Chiang et al. explained these effects through EMT blocking mediated by the downregulation of Snail, Slug, Vimentin, and the cadherin switch [89]. Li et al. showed that FoxM1, and consequently β-catenin suppression, might participate in VD’s inhibition of growth, migration, invasion, and stemness [107].

**Table 5:** Preclinical studies evaluating the effect of VD or VDAs on in vitro and in vivo models of PDAC (from Pubmed indexed articles, accessed on 1 May 2024).

Ref.	Substance Investigated	Model	Investigation Techniques	Results	Mechanism
[111]	22-oxa-calcitriol Calcitriol	In vitro— human PDAC cell lines In vivo— mouse xenografts	Proliferative assays. Tumor volume in xenografts. Cell cycle analysis with flow cytometry.	In vitro, 22-oxa-calcitriol and calcitriol inhibited proliferation and caused a G1 phase cell cycle arrest. In vivo, 22-oxa-calcitriol had a more potent anti-tumor growth effect than calcitriol without inducing hypercalcemia.	Antiproliferative effect mediated by G1 cell cycle arrest.
[112]	Seocalcitol (EB 1089)	In vitro— human PDAC cell lines	Proliferative assays.	EB1089, if added alone to the cells, did not significantly inhibit growth. Combined with all-trans-retinoic acid, an anti-tumor growth effect of 90% in Capan 1 cells and of 70% in Capan 2 cells was observed.	Antiproliferative effect in association with retinoids.
[98]	22-oxa-calcitriol Calcitriol	In vitro— human PDAC cell lines	Proliferative assays. Cell cycle analysis with flow cytometry. Levels of expression of cyclins (D1, E and A), cyclin-dependent kinases (2 and 4), and cyclin-dependent kinase inhibitors (p21 and p27) were analyzed via Western blotting.	Marked upregulation of p21 and p27 after 24 h treatment and marked downregulation of cyclins and CKD after 7 days of treatment were observed.	Antiproliferative effect mediated by upregulation of CDK inhibitors (p21 and p27), which in turn blocks G1/S transition.
[95]	Seocalcitol (EB 1089)	In vitro— human PDAC cell lines In vivo— mouse xenografts	Proliferative assays. Tumor volume in xenografts. Western blotting for p53 levels.	EB 1089 had a dose-dependent anti-tumor growth effect in vitro and in vivo. No appreciable difference in p53 protein levels relative to controls was observed.	Antiproliferative effect independent of p53.
[109]	Seocalcitol (EB 1089) 20-epi-vitamin D3 (CB 1093)	In vitro— human PDAC cell lines	Proliferative and apoptosis assays.	VDAs inhibited tumor growth. Increased proportion of cells in the G0/G1 phase were observed after VDA-treated media. Neither induced apoptosis.	Antiproliferative effect mediated by G1 cell cycle arrest.
[97]	Seocalcitol (EB 1089)	In vitro— human PDAC cell lines	Proliferative assays.	VDA treated cell lines presented decreased cell numbers.	Antiproliferative effect.
[96]	Calcidiol Calcitriol	In vitro— human PDAC cell lines, KRAS transfected human PDAC cell lines	Proliferative asssays. Cell cycle analysis with flow cytometry. CDK inhibitors (p21 and p27) were analyzed by Western blotting.	VD inhibited tumor growth, upregulated CDK inhibitors p21 and p27 and induced cell cycle arrest at G1/S checkpoint proportionally. Tumor growth was also inhibited in mutant-K-Ras-transfected and endogenous K-Ras-activating mutation cell lines.	Antiproliferative effect mediated by upregulation of CDK inhibitors (p21 and p27), which in turn blocks G1/S transition. Ki-Ras mutations do not interfere with the growth-inhibitory effects of VD.
[113]	Seocalcitol (EB 1089)	In vitro— human PDAC cell lines	Proliferative assays.	VDAs decreased the PC cell number.	Antiproliferative effect.
[98]	Maxacalcitol (22-oxacalcitriol) Calcitriol In vitro— human PDAC celllines In vivo— mouse xenografts	In vitro— human PDAC celllines In vivo— mouse xenografts.	Proliferative assays. Tumor volume in xenografts. Cell cycle analysis with Flow cytometry. Levels of p53 determined via Western blotting.	In vitro, VDAs inhibited proliferation and caused G1 Phase cell cycle arrest. In vivo, maxacalcitol Inhibited the growth of BxPC-3 xenografts more significantly than calcitriol, without inducing hypercalcemia. Inresponsive cells, p21 and p27 Were markedly upregulated after treatment with Both agents. In non-responsive cells, no such changes were observed.	Antiproliferative Effect mediated by CDK inhibitors (p21 and p27) upregulation, which in turn leads to G1 cell cycle arrest.
[110]	Seocalcitol (EB 1089)	In vitro— human PDAC cell lines	Proliferative and apoptosis assays. Colorimetric assay based on cleavage of a caspase-associated substrate. Immunoblotting for determing protein substrate.	VDA alone and in association with retinoic acid (9-cis RA) significantly inhibited cell growth. VDA diminished apoptosis induced by 9-cis RA in tumor cells via caspase inactivation. EB1089 upregulates p21, but not p27. Akt phosphorylation is not reduced by EB1089.	Antiproliferative effect. Anti-apoptotic effect exerted at a level upstream of caspase activation.
[100]	Paricalcitol (19-nor-calcitriol) Calcitriol	In vitro— human PDAC cell lines In vivo— mouse xenografts	Proliferative asssays. Tumor volume in xenografts. Cell cycle analysis with flow cytometry. CDK inhibitors (p21 and p27) were analyzed via Western blotting.	VDAs inhibited cell proliferation in 3 out of 4 lines. The effect was dose-dependent and correlated with increased levels of CKD inhibitors, p21 and p27. Tumor inhibition was accompanied by in vivo upregulation of p21 and p27 expression.	Antiproliferative effect mediated by upregulation of CDK inhibitors (p21 and p27), which in turn leads to G1 cell cycle arrest.
[105]	Calcitriol-3 -bromoacetate	In vitro— human PDAC cell lines	Proliferative asssays. P21, p53, and Akt were analyzed via Western blotting.	VDA inhibited cell growth and upregulated p21 levels, with no effect on p53. Weakly inhibited Akt phosphorylation.	Antiproliferative effect, possibly via Akt/PI3K pathway.
[103]	Calcitriol	In vitro— human PDAC cell lines, transfection for Smo, Ptch1, VDR In vivo— mouse xenografts	Proliferative asssays. Tumor volume, metastasis and mortality in mouse xenografts. Western blotting.	In vitro, VD inhibited cell growth through inactivation of Smo and the downstream Hh pathway, rather than through VDR activation. In vivo, VD was ineffective.	Antiproliferative effect in vitro, partly mediated by Hh inactivation (by directly inhibiting Smo, independently of VDR binding).
[104]	Calcitriol	In vitro— human PDAC cell lines In vivo— mouse xenografts	Proliferative and apoptosis asssays. Tumor volume in xenografts. Caspase activity assay. Regulation of protein levels and posttranslational events were detected using Western blotting.	Calcitriol promotes gemcitabine antiproliferative effect and apoptotic effect. Alone, VD had minimal effect on caspase activation—modest activation of caspase-9 but not that of caspases-8, -6 or -3. However, it enhanced the caspase activity when used in combination with gemcitabine. Calcitriol slightly decreased the level of phosphorylated Akt, which was further reduced following the addition of gemcitabine. In vivo, Calcitriol alone resulted in tumor regrowth delay compared with saline control in a dose-dependent manner. The combination treatment further inhibited tumor growth with a 10-fold reduction in tumor size (*p* < 0.01).	Antiproliferative effect, possibly via Akt/PI3K pathway. Calcitriol promotes gemcitabine-mediated caspase-dependent apoptosis.
[101]	19-nor-2α- (3-hydroxypropyl)- calcitriol (MART-10) Calcitriol	In vitro— human PDAC cell lines In vivo— mouse xenografts	Proliferative and apoptotic assays. Tumor volume in xenografts. Cell cycle analysis with flow cytometry.	Antiproliferative effect in vitro and in vivo. G0/G1 cell cycle arrest. Upregulation of p21 and p27. No apoptotic induction.	Antiproliferative effect by upregulation of CDK inhibitors (p21 and p27), which in turn leads to G0/G1 cell cycle arrest.
[102]	Calcitriol	In vitro— human PDAC cell lines	Proliferative assay. Western blotting for determing p21.	Calcitriol inhibited proliferation of PC cells and is involved in the upregulation of CDK inhibitor p21.	Antiproliferative effect mediated by upregulation of CDK inhibitor p21.
[89]	19-nor-2α-(3-hydroxypropyl)- calcitriol (MART-10) Calcitriol	In vitro— human PDAC cell lines	Migration and invasion assays. Regulation of protein levels and posttranslational events were detected using Western blotting.	1α,25(OH)2D3 and MART-10 repressed migration and invasion in vitro, inhibited EMT through the downregulation of Snail, Slug, and Vimentin expression, blocked cadherin switch (from E-cadherin to N-cadherin). Both decreased MMP-2 and -9 secretion and F-actin synthesis.	Cell migration and invasion inhibition by blocking EMT process, which was supported by downregulation of Snail, Slug, as well as Vimentin, and cadherin switch. Repression of secretion of MMP2 and MMP-9 and F-actin synthesis in the cytoplasm.
[107]	Seocalcitol (EB 1089) Calcitriol	In vitro— human PDAC cell lines, human tissue microarrays In vivo— mouse xenografts	Lentiviral VDR expression vector construction and transfection. Proliferation, migration, invasion, and spheroid colony formation assays. Tumor volume in xenografts. Immunohistochemestry (IHC) for tissue samples. Western blot for protein levels.	Inverse correlation between reduced expression of VDR and increased expression of FOXM1. 1,25VD and EB 1089 inhibited FOXM1 and b-catenin expression and suppresed growth, migration, invasion and spheroid formation of PC cells.	Antiproliferative effect, cell migration, invasion and stemness inhibition, possibly by repressing FOXM1 signaling.
[114]	Calcitriol Poly- lactic-co-glycolic acid (PLGA) nanoparticles Free Calcitriol	In vitro— human PDAC cell lines	Proliferative and cytotoxic assays. Cell cycle analysis with flow cytometry. Laser scanning confocal microscopy for evaluation of nanoparticle uptake.	Encapsulated calcitriol enhanced its inhibitory effect on cell growth compared to free calcitriol. Cell cycle analysis demonstrated a significant accumulation of both pancreatic cell lines in the G0/G1.	Antiproliferative effect mediated by G0/G1 cell cycle arrest
[106]	Calcipotriol	In vitro— hPDAC cell lines	Proliferative assay. Western blotting, rtPCR. Gene knockdown. Microarray data analysis	PDAC growth inhibition mediated by calcipotriol is linked to VDR expression and Wnt signaling. Calcipotriol reduces LRP6 protein levels. LDLRAP1 mediates reduced LRP6 protein levels in response to calcipotriol. VDR is regulated by Wnt signaling.	Antiproliferative effect mediated by autocrine inhibition of Wnt/β-catenin signaling through regulation of LRP6 and LDLRAP1.
[115]	Calcidiol	In vivo— mouse xenografts	MRI to assess tumor volume in mice with deficient/control/excess dietary VD.	No measurable impact of dietary VD was found on PDAC development, growth, or mortality.	
[78]	Calcidiol	In vitro— hPSCs extracted from PDAC biopsies Co-cultures of hPSCs and PDAC cells	hPSC extraction from biopsies. IHC for aSMA. Invasion assay. Cell viability asssay.	hPSCs were activated by irradiation. In VD-treated media, aSMA expression in irradiation-activated hPSCs was decreased compared to controls. In co-culture experiments, PDAC cell viability and invasion capacity were also decreased in the VD-treated group.	Inhibition of stromal cell activation.
[116]	Calcipotriol	In vitro— hPDAC cell lines, hPCSs cell line In vivo— Mouse orthotopic allografts and KPC engineered model, both assigned to treatment groups: gemcitabine, calciptriol, or the combination	RNA-Seq to assess the genomic effects of VDR activation in PSCs. Ultrasound to assess tumor volumes in mice. Quantification of intratumoral drug concentration.	VDR activation promotes the quiescent PSC state and inhibits tumor-supportive pathways. In allograft models, VD decreased stromal activation and fibrosis. In both mouse models, combination therapy reduced tumor volume, increased intratumoral vasculature and intratumoral concentration of gemcitabine, and survival was prolonged compared to gemcitabine alone.	Prevention of PSC activation, hindering stromal remodeling. Promotes gemcitabine efficacy by increasing its intratumoral concentration.
[117]		In vitro— hPDAC cells	RNA seq. WB. Cell viability assays. Plasmids.	Compared to control, only 8% of VDR-knockdown cells survived after gemcitabine treatment. VDR knockdown delayed foci formation and reduced foci intensities of phospho-gH2AX and Rad51.	VDR activation is implicated in DNA repair through Rad51 recruitment to sites of DNA damage. VDR depletion enhances gemcitabine sensitivity.
[79]	Seocalcitol (EB 1089)	In vitro— hPDAC cell lines	Cell cycle analysis. Apoptosis assay. Protein extraction. WB.	VDA’s antiproliferative effect on PC cells depended on VDR expression. VDA treatment increased the G1 population and decreased S and G2 populations. Apoptotic rate also increased after VDA treatment. β-Catenin and phosphorylated Akt expression decreased, but p21 expression increased in VDA-treated PC cells. Also, Bcl-2 expression decreased, but Bax expression increased in VDA treated PC cells.	Antiproliferative effect mediated by upregulation of CDK inhibitor p21 leading to G0/G1 cycle arrest and by β-catenin and Akt pathways downregulation. Apoptotic effect on PC cells by Bax/Bcl-2 pathway deregulation.
[118]	Calcitriol	In vitro— human normal pancreatic cell lines and poorly differentiated metastatic PDAC line Panc-1.	Western blot.	VD alters the VDR level in normal pancreatic cells but not in PC cells. High concentration downregulates VDR. VD increased p21 expression and induced early expression of CKD1 in normal pancreatic cells. These effects were not seen in PC model.	Poorly differentiated metastatic PC line Panc-1 is resistant to VD effects.
[119]	Calcitriol	In vitro— human PDAC and CAF cell lines	Fluorescence microscopy. Flow cytometry. RNA-seq. CRISPR/CAS9 system. RT-PCR. Migration and invasion assay.	VDR activation on CAFs significantly decreased the exosome-induced upregulation of miR-10a-5p in PC cells. MiR-10a-5p deletion decreased CAF’s promigratory and proinvasive effects on PC cells. The effect could be rescued by enforced expression of miR-10a-5p in PC cells.	Tumor migration and invasion inhibition mediated by suppressing CAF secretion of exosomal oncomicroRNA miR-10a-5p.
[120]	Calcitriol	In vitro— PDAC cell lines (2D and 3D) In vivo- Mouse xenografts	RNA sequencing. qPCR. Migration assay. Tumor volume. Survival analysis.	VD increased E-cadherin expression in epithelial type PC cells (E). In quasi-mesenchymal (QM) PC cells, VD increased fibronectin 1 expression. VD enhanced migration in 3D models of MQ cells, but not in E cells models. In vivo models showed that VD increased metastasis generation in MQ tumors. The tendency was reversed in E tumors.	VD has different effects on PC subtypes: - Increasing epithelial phenotype and reducing metastasis propensity in E tumors - Opposite effects in MQ tumors
[82]		In vitro— hPDAC cell lines	Cell transfection. qPCR. WB. Proliferation, migration and invasion assays.	Compared to controls, in the CYP24A1-silenced PDAC cells, cell proliferation and invasion decreased (*p* = 0.035, respectively *p* = 0.002). No effect on cell migration ability was noted.	CYP24A1 silencing decreases proliferation and invasion of PDAC cells.
[121]	Calcipotriol	In vitro— hPDAC cell line BxPC3 (SMAD4 deficient) and BxPC3-SMAD4+ Human peripheral blood mononuclear cells (PBMCs) in PDAC cell line CM	Isolation of PBMCs. Immunoblot and intracellular calcium flow analysis. Cytokine and T-lymphocyte proliferation assay. Flow cytometry.	VDA increased intracellular calcium accumulation in non-conditioned and BxPC3-SMAD4+ conditioned PBMCs, but not in BxPC3-conditioned ones. VDA reduces TNF-α release by PBMCs, but increases TGF-β release. VDA did not inhibit PDAC-induced lymphocyte proliferation, but antagonized PDAC-induced apoptosis. VDA partially upregulated PDAC-inhibited NF-κB signaling pathway.	Counteracts PDAC-induced alterations in TME inflammatory cells (intracellular calcium increase, TGF-β release, antiapoptotic).
[122]	Calcipotriol	In vitro— hCAFs and hPDAC cell lines 2D or 3D cultures	Contraction, scratch wound healing, and proliferation assays. RT qPCR. ELISA. Flow cytometry.	VDA decreased proliferation and migration of CAFs and reduced the release of their pro-tumorigenic factors, such as prostaglandin E2, IL-6, periostin, and leukemia inhibitory factor. However, it promoted upregulation of PD-L1 in CAFs. VDA reduced activation and production of IFN-γ, granzyme B, and IL-17 by T cells, in an additive manner with CAFs. VDA inhibited tumor-inflitrating lymphocytes in PC model.	Dual antagonistic effect in PDAC: - CAFs pro-tumorigenic effect inhibition - T cell immunity reduction.
[123]	57 nonsecosteroidal VDA with phenyl-pyrrolyl pentane scaffold	In vitro— hPSC (2D and 3D) and hPDAC cell lines In vivo— Mouse xenograft with co-incoluation of PSC and PDAC cell lines	VDR binding, transcription, proliferative, anti-PSC activity and cell survival assays. RT qPCR. WB. IF. Imaging for tumor volume.	VDAs suppressed collagen I and α-SMA expression in PSC cultures. PC cell proliferation in PSC supernatant medium was significantly decreased when VDA as added. VDA treatament increased doxorubicine penetration in PSC spheres. C4, 15, and 18 VDA plus gemcitabine significantly reduced tumor volume in vivo.	Inhibition of: - PSC activation - ECM deposition - PSC-induced PDAC cell proliferation. Tumor volume reduction in combination with gemcitabine.
[124]	Calcitriol (in combination with 13-cis retionic acid)	In vitro— hPDAC cell lines	Cell survival and invasion assays. Protein extraction. qPCR. WB.	In PDAC cell lines treated with TNFα, combination treatment: mildly increased E-cadherin, decreased N-cadherin and nuclear levels of Slug and Twist1; inhibited cell invasion; reduced MMP-9 mediated by inactivation of JNK and NF-κB signaling pathways; increased TIMP3 through miR-221 supression.	Combination treatment counteracts TNFα-mediated effects: - Modulation of EMT-regulatory proteins - Inhibition PC cell invasion - Inhibition of JNK and NF-κB signaling pathways, reducing MMP-9 - Upregulation of TIMP-3 through of JNK pathway inhibition and miR-221 downregulation.
[125]	Calcitriol	In vitro— hPDAC cell lines	Protein extraction. qPCR. WB.	TGFβ increased VDR expression. When added to PDAC cell lines treated with TGFβ, VD reverted E-cadherin and fibronectin expression back to levels similar to the control.	Counteracts TGFβ-induced EMT
[126]	Calcipotriol	In vitro— hPDAC cell lines, 3D organotypic cultures In vivo— Mouse xenografts *both treated with recombinant orthopoxvirus CF33	Viral growth assay. Protein extraction. WB. Tumor volume. IF. IHC.	VDA had no direct inhibitory effect on viral replication. In 3D organotypic cultures, VDA decreased myofibroblast integrity. In VDA-pretreated mice, increased CF33 tumor penetrance and replication were observed. Increased vascular lumen size and reduced FoxP3+ regulatory T cells were noted in VDA+CF33-treated tumors.	VDA induced stromal modifications and increased virus penetration in the tumor.
[127]	Calcipotriol	In vitro— hPCS and PDAC cell lines, PSC/PDAC 3D co-cultures In vivo— Genetically engineered PDAC mouse models	Calcipotriol-loaded lysosomal pH-buffering (C-LBM) and non-pH-buffering micelles (C-NBM). Cell uptake, intracellular trafficking, lysosomal pH, and lysosomal degradation assays. Cell viability assay. Lipid droplet accumulation assay. WB. qPCR. Tumor volume. Histopathology studies. Flow cytometry.	LBM, an inhibitor of autophagy, decreased α-SMA levels and increased lipid droplets in PSCs, suggesting PSC deactivation. VDA alone slightly downregulated α-SMA levels and enhanced lipid droplets, in WT-PSC cell lines, but not in p62-deficient PSC. C-LBM had a marked effect on PSC deactivation and decreased the expression of protumoral cytokines (IL6, TGFβ) in PSCs. C-LBM decreased PSC-induced proliferation of PDAC cells and increased gemcitabine penetration in 3D PSC/PDAC co-cultures. In vivo, C-LBM deactivated PSCs, diminished fibrosis, increased vascular density and vessel diameter, enhanced gemcitabine deliver, y and increased the Teff/Treg ratio in the TME. Combination therapy of C-LBM + gemcitabine + antiPL1 in PDAC mice showed a 2-fold increase in the median survival time.	PSC activation leads to increased autophagy and reduced p62 levels, desensitizing VDR signaling. VDAs associated with autophagy inhibitors allow proper VDR signaling, leading to PSC deactivation with reduction in fibrotic and immunosuppressive components of the stroma.
[128]	Calcitriol Calcipotriol Paricalcitol	In vitro— hPCS and PDAC cell lines, In vivo— Mouse xenografts	Plasmid transfection and siRNA. Cell viability assay. Reverse-phase protein array (RPPA). qPCR. WB. IF and IHC. Tumor volume. Mass spectrometry.	VD/VDA induced VDR protein expression in PDAC cells. VD/VDA inhibited PDAC cell growth in vitro. RRPA showed that MUC1 was signifcantly downregulated by VD/VDA (CD44, FoxM1, and GLUT1 expression were also inhibited). VDR overexpression dose-dependently inhibited MUC1 expression in vitro, while VDR knockdown increased protein presence. Treatment with VDA or activation of VDR gene suppressed MUC1 levels. VDR-knockdown PDAC cells had increased gemcitabine resistance. VDA treatment increased drug sensitivity. In MUC1 knockdowns, gene transduction significantly increased drug sensitivity. In vivo, VDA + gemcitabine significantly inhibited tumor growth (*p* = 0.016), while treatment with gemcitabine or VDA alone did not reach statistically significant tumor reduction.	Positive feedback between VD and VDR expression. VD-VDR downregulates MUC1 expression. VD-VDR activation increases gemcitabine sensitivity, and MUC1 downregulation plays a role in this pathway.
[108]	Calcipotriol	In vitro— hPDAC cell lines	Cell proliferation, viability, and migration assays. Protein isolation and immunoblotting. RNA isolation. qPCR. Molecular cloning and virus transfection.	VD-resistant PC cell lines presented reduced expression of VDR proteins and mRNA. Low H3K27ac occupancy in the promoter region of the VDR gene was noted in those PC cell lines. Treatment with glyceryl triacetate increased H3K27ac and VDR protein levels and slightly sensitized cells to VD. Similar results were seen with 5Aza, a drug with DNA methylation reduction properties. Combination treatment increased VD antiproliferative activity more significantly compared to each treatment alone, most probably by upregulating tumor suppressor genes (CDKN1A or p21, DUSP10, NOG) and downregulating oncogenes (DOCK1, EFNA1, Hey1, and TGFB2). Addition of VDA increased these effects.	VDR is silenced at a transcriptional level in VD-resistant PC cell lines. This may be caused by H3K27 hypoacetylation or DNA methylation. Reversing these processes increases the antiproliferative effect of VD by modifying PDAC cells’ gene transcription profile.
[81]		In vitro— human PDAC cell lines and co-cultures with macrophages In vivo— Mouse xenografts Ex vivo— human PC tissue from 87 patients with no prior theraphy	WB. ELISA. ChIP. Dual-luciferase reporter assays. Migration assay. Tumor volume. IF. IHC. Flow cytometry.	VDR overexpression in conditioned medium stimulated M0 macrophages to differentiate into M2 lineage. VDR overexpression increased CCL20 levels in WT cells, but not in CCL20-promoter-knockdown ones. Using CCL20-neutralizing antibodies in the conditioned medium reduced M2 recruitment. Human PDAC tissue with higher VDR expression had higher M2/M1 ratio in both the tumor and stroma. In vivo, CCL20 blocking had antiproliferative effects and reduced M2/M1 ratio.	By binding to CCL20 promoter, VDR activates gene transcription. In turn, CCL20 promotes macrophage differentiation into M2 phenotype. CCL20 blockade presented antiproliferative effects.

A limitation of the aforementioned studies is that they investigated the effect of VD solely on tumor cell populations and did not account for the interaction with the TME. To overcome this limitation, VDR expression in stromal cells was first demonstrated [78,116]. Subsequently, different study designs were envisioned to better simulate the in vivo conditions. Mukai et al. used co-cultures of PDAC cells and irradiation-activated PSCs. By treating the samples with VD, they observed that PSC activation (evaluated based on α-SMA expression and lipid droplet content) and PSC-induced invasion and survival of PDAC cells decreased compared to the controls, concluding that VD inhibits stromal activation [78]. Sherman et al. reported similar results, observing that VD promotes the reversal of activated PSCs to their quiescent state. In addition, using the MIAPaCa-2 cell line, a human PDAC cell line known to have low VDR expression, cultured in conditioned media (CM) from activated PSCs, Sherman et al. showed that VDR activation of the stroma (and not epithelial PDAC cells) induces genomic modifications in tumor cells, altering the expression of genes involved in proliferation, survival, EMT, and chemoresistance [116]. Moreover, using allograft models of VD-resistant PDAC cell lines, the authors found that VD reduces stromal activation and fibrosis. In both allograft and engineered mouse models, VD treatment enhanced gemcitabine efficacy by increasing the intratumoral vasculature and drug concentration [116]. Kang et al. also found that VD inhibits activated PSCs in vitro and enhances gemcitabine efficacy in vivo [123]. Kong et al. observed that in activated PSCs, VDR inhibition was impaired owing to autophagy-induced p62 loss. Coupling VDA with an autophagy inhibitor, they noted increased PSC inactivation, the downregulation of pro-tumorigenic factors, the inhibition of PDAC cell PSC-induced proliferation, and increased gemcitabine penetration in 3D PSC/PDAC co-cultures [127].

Kong et al. demonstrated that VD also alters the interaction between the stroma and PDAC cells by decreasing exosomal miR-10a-5p production and release by CAFs. miR-10a-5p is known to be associated with PDAC [119].

The role of VD in the crosstalk between PDAC cells and the TME is not well understood. Using peripheral blood mononuclear cells in the PDAC cell line CM, Moz et al. demonstrated that VD inhibits tumor cell-induced apoptosis of immune cells in a SMAD-dependent manner (when using SMAD-deficient PDAC cell line CM, the effect was not significant) [121]. Unexpectedly, Gorchs et al. found that VD might hinder effector T lymphocyte immunity. They observed that even though VD reduced CAF migration, proliferation, and the upregulation of pro-tumorigenic factors, it also increased PD-L1 expression and reduced T cell activation and proliferation [122]. In vivo experiments conducted by Kong et al. noted that VDA in combination with an autophagy inhibitor (added to increase the PSC response to VDA) increased the Teffector/Tregulatory ratio in the TME. Combining VDA and the autophagy inhibitor with gemcitabine and an anti-PL1 agent resulted in a 2-fold increase in median survival in PDAC mouse models [127]. Recently, Li et al. showed that VDR promotes macrophage TME infiltration and differentiation into the M2 phenotype through CCL20 signaling [81].

To sum up, to our current understanding, VD can act on PDAC cells either directly, inhibiting proliferation, apoptosis, EMT, migration, invasion, and stemness, or indirectly by returning activated PSCs to their quiescent state. Inactivated PSCs alter the pro-tumorigenic cross-talk between components, decreasing the PSC-induced survival, proliferation, and invasion of PDAC cells and inducing stroma remodeling (reducing fibrosis and increasing stromal vasculature), leading to better drug penetration. However, the results regarding the effect of VD on the immune component of the TME are conflicting. While some studies showed decreased immune cell apoptosis and an enhanced stromal T effector/T regulatory ratio in response to VD, others found the upregulation of PD-L1 expression, reduced effector T cell activation and proliferation, and increased macrophage stromal infiltration and polarization.

### 3.6. Clinical Trials of VDAs in PDAC

Based on the observed effects of VD in preclinical studies, multiple clinical trials were designed in order to capitalized on these benefits.

To overcome VD-induced hypercalcemia, most clinical trials use synthetic analogs designed to have a low calcemic effect while retaining non-classical actions (see Table 6).

A phase II single-group trial comprising 36 patients with inoperable PC (the study included other PC histopathological subtypes apart from PDAC) who received escalating high doses of seocalcitol noted satisfactory tolerability of the drug, but did not observe any improvement in the outcome (assessed based on time to treatment failure [TTF] and OS) [129]. Another phase II single-group trial including 25 participants with inoperable PDAC treated with docetaxel and calcitriol found a modest increase in TTP compared to previously reported data on docetaxel treatment only [130]. A more recent open-label pilot study investigated paricalcitriol (either fixed or weight-based doses) combined with nanoliposomal irinotecan and 5-fluorouracil/leucovorin in 20 patients with inoperable PDAC that progressed or were intolerant to gemcitabine chemotherapy. Both regimens were safely tolerated. The arm with weight-based doses, where higher doses could be achieved, presented with increased tumor vascularity [131].

There are multiple clinical trials on VDA treatment in PDAC registered on Clinicaltrials.gov, either completed or ongoing, with results yet to be published (Table 7).

To reconcile the conflicting results regarding serum vitamin D (VD) levels and their association with PDAC risk and prognosis, several approaches can be proposed. Mendelian randomization studies offer a robust method to establish causality by leveraging genetic determinants of VD levels to assess their association with PDAC, thereby minimizing the impact of confounding variables and reverse causality. Standardized measurement protocols are essential to reduce variability across studies, ensuring the use of uniform and reliable methods, such as liquid chromatography-tandem mass spectrometry, to accurately quantify serum VD levels. Additionally, stratified analyses are critical for elucidating population-specific trends by examining subgroups based on factors, such as age, gender, and ethnicity, which may influence VD metabolism and cancer biology. Furthermore, integrating mechanistic studies can enhance the understanding of VD’s role in PDAC by exploring its effects on tumor–stroma crosstalk, immune responses, and gene expression. These approaches collectively aim to address the limitations and heterogeneity inherent to existing studies, providing a clearer perspective on the relationship between VD and PDAC.

It is highly likely that the observed effects of vitamin D (VD) on the progression of pancreatic ductal adenocarcinoma (PDAC) are influenced by additional cofactors, such as genetic predisposition and environmental influences. Genetic variations in key VD-related genes, including VDR, CYP27B1, and CYP24A1, may alter VD bioavailability, receptor binding affinity, and downstream signaling pathways. For instance, the Fok1 single-nucleotide polymorphism (SNP) in the VDR gene modifies receptor activity and has been associated with variable PDAC risks in different populations. Furthermore, the genetic heterogeneity of PDAC tumors, driven by mutations in genes, such as KRAS, TP53, CDKN2A, and SMAD4, may significantly influence how VD impacts tumor cell proliferation, apoptosis, epithelial-to-mesenchymal transition (EMT), and other cancer hallmarks [108].

Environmental factors further modulate the effects of VD on PDAC. Variability in UV-B exposure, dietary habits, and supplementation practices can significantly influence baseline VD levels, leading to inter-population differences in responsiveness to VD interventions. Additionally, the composition of the tumor microenvironment (TME), including stromal elements and immune cell infiltration, is shaped by environmental influences and plays a critical role in determining VD’s effects. For example, VD has been shown to modulate stromal activation and immune cell responses in preclinical studies, but the extent and nature of these effects may depend on the unique TME characteristics of each patient [83].

The complex interaction between vitamin D (VD) and the tumor microenvironment (TME), particularly its effects on cancer-associated fibroblasts (CAFs), significantly influences the clinical applicability of findings in pancreatic ductal adenocarcinoma (PDAC). VD’s ability to inactivate CAFs and remodel the stroma, enhancing drug penetration and reducing tumor-promoting cross-talk, is promising. However, the heterogeneity of CAF subtypes, with both tumor-promoting and tumor-suppressing roles, complicates predicting VD’s net effect. Additionally, VD’s variable influence on immune responses, such as altering PD-L1 expression or T cell activity, further highlights the need for tailored approaches. Future clinical applications should focus on individualized TME profiling and advanced preclinical models to ensure the precise and effective use of VD in PDAC therapy [132].

These complex interactions highlight the need for careful interpretation of study results. The observed effects of VD on PDAC may not solely reflect VD’s intrinsic properties but instead represent the cumulative influence of genetic and environmental factors. To address this, future studies should incorporate stratified analyses to evaluate the effects of VD within subpopulations defined by genetic and environmental characteristics. Additionally, controlled study designs, such as Mendelian randomization, can help disentangle VD’s direct effects from confounding variables. By accounting for these cofactors, research can better identify patients most likely to benefit from VD-based therapeutic strategies and refine our understanding of VD’s role in PDAC progression.

The main limitations of this systematic review primarily stem from the significant heterogeneity among the included studies. Variability in study designs, ranging from observational epidemiologic studies to preclinical and clinical trials, makes direct comparisons challenging. Observational studies often relied on proxies for vitamin D levels, such as UV-B exposure or dietary intake, which may not accurately reflect biologically active vitamin D concentrations. Serum vitamin D measurements, while more precise, were conducted using varying methodologies, such as immunoassays or liquid chromatography-tandem mass spectrometry, with differing levels of accuracy and reliability. Furthermore, the timing of vitamin D measurements (e.g., seasonal variations or single time-point assessments) and inconsistencies in the categorization of vitamin D levels into arbitrary “high” or “low” groups introduce additional variability.

Population heterogeneity also poses a significant limitation. The included studies encompass diverse demographic groups with differences in age, gender, ethnicity, and geographic location, all of which influence vitamin D metabolism and its potential effects on pancreatic ductal adenocarcinoma (PDAC). These factors, combined with varying exposure to environmental influences, such as sunlight, dietary habits, and supplementation, further complicate the interpretation of results. Additionally, genetic predispositions, including polymorphisms in vitamin-D-related pathways, were not consistently accounted for, which could impact both vitamin D metabolism and its interaction with the tumor microenvironment.

Finally, the heterogeneity in endpoints across studies limits the ability to draw cohesive conclusions. Some studies focused on PDAC risk, while others examined progression, survival, or tumor response to therapy. Variability in the definitions and measurements of these endpoints further contributes to inconsistencies. Confounding factors, such as treatment-related vitamin D deficiencies, comorbidities, and differences in the tumor microenvironment composition, were often inadequately controlled, raising questions about causality versus correlation. These limitations emphasize the need for more standardized study designs, uniform measurement methodologies, and detailed subgroup analyses in future research to clarify the role of vitamin D in PDAC.

Future research to clarify the relationship between vitamin D (VD) and pancreatic ductal adenocarcinoma (PDAC) should focus on addressing key limitations and resolving conflicting evidence. First, standardized measurement techniques are essential to ensure consistency across studies. Utilizing accurate methods, such as liquid chromatography-tandem mass spectrometry, and conducting longitudinal studies with repeated measurements will provide a better understanding of long-term VD exposure. Additionally, stratified analyses based on demographics, including age, gender, ethnicity, and genetic predispositions, such as polymorphisms in VD-related genes, like VDR and CYP27B1, are needed to identify population-specific associations and responses.

To establish causality, Mendelian randomization studies can leverage genetic markers associated with VD metabolism to isolate their direct impact on PDAC risk and progression while minimizing the influence of confounding factors. Advanced preclinical models, such as patient-derived xenografts or organoid systems, should be employed to simulate human tumor microenvironments, enabling detailed investigations of VD’s effects on tumor cells, stromal interactions, and immune modulation. In clinical trials, integrating comprehensive tumor profiling will be critical to evaluate how VD affects specific components of the tumor microenvironment, including cancer-associated fibroblasts (CAFs) and immune cells, and to identify biomarkers predictive of the therapeutic response.

Mechanistic studies should explore how VD influences key aspects of tumor–stroma cross-talk, such as the behavior of CAF subtypes, immune responses, and extracellular matrix remodeling, to determine the conditions under which VD exerts tumor-suppressive versus neutral or pro-tumorigenic effects. Finally, a personalized approach is vital, tailoring VD-based interventions to the unique tumor microenvironment and genetic profile of individual patients. This will help ensure that therapies are targeted and effective, ultimately advancing our understanding of VD’s potential role in PDAC prevention and treatment.

## 4. Conclusions

Mechanistic studies provided us with grounds for optimism regarding the potential of VD as a therapeutic agent in PDAC treatment. Initially, interest was generated by VD’s direct action on tumor cells, inhibiting proliferation, apoptosis, EMT, migration, invasion, and stemness. Subsequently, the indirect effect of VD on PDAC cells through stroma remodeling caught the attention of the research community. Following the unexpected results of increased tumor aggressiveness after stromal depletion, the focus shifted towards finding a stromal inactivation agent, and VD, with its role of returning activated PSCs to their quiescent state, reversing pro-tumorogenic cross-talk between components, reducing ECM stiffness, and enhancing the stromal Teffector/Tregulatory ratio, seemed to fit the description perfectly. However, are these results sufficient to move VD and VDAs from bench to bedside? Should enthusiasm be tempered by the conflicting results of epidemiological studies despite their shortcomings? Do VD’s direct anticarcinogenic effects observed in simple PDAC cell cultures or in tumor models of immunocompromised mice with reduced stromal reaction also occur in human PDAC tissues surrounded by the complex TME? Should the promising effects of VD on PCSs disregard the variability within CAF populations, for which actions range from tumor-limiting to tumor-promoting, and how these may be affected by VD? Currently, it seems that VD may act as a modulator of the intricate tumor–stroma crosstalk.

From a theoretical perspective, the next important questions to answer are: (1) how the TME composition influences the natural evolution of PDAC, (2) how VD acts on different CAF populations, and (3) what is the net result of VD on PDAC when each individual effect is added? Understanding these aspects would ideally allow us to use VD and VDAs in a targeted manner, tailored to the individual TME composition. From a more practical standpoint, what we consider achievable at this stage to narrow the gap toward a personalized VD therapy is creating more reliable preclinical PDAC models, reducing the cost of tumor-profiling techniques in order to allow for clinical implementation, and continuing the more empirically, but more result-oriented investigation of VD’s effects through well-controlled clinical trials.

## Figures and Tables

**Table 2:** Methodology differences in studies assessing VD levels and PDAC risk.

Ref.	Technique for Quantitative Determination of VD Levels	Approach Used for Dividing the Population by Means of VD Level
[41]	Radioimmunoassay	Quartiles
[38]	Radioimmunoassay	Quartile
[43]	Direct Competitive Chemiluminescent Immunoassay	Intervals set as <25 nmol/L, 25–37.5 nmol/L, 37.5–50 nmol/L, 50–75 nmol/L, 75–100 nmol/L, ≥100 nmol/L
[39]	Enzymeimmunoassay	Intervals set as <50 nmol/L, 50–75 nmol/L, ≥75 nmol/L
[44]	Radioimmunoassay or Direct Competitive Chemiluminescent Immunoassay	Quartiles
[15]	Direct Competitive Chemiluminescent Immunoassay	Intervals set as <25 nmol/L, 25–37.5 nmol/L, 37.5–50 nmol/L, 50–75 nmol/L, 75–100 nmol/L, ≥100 nmol/L
[37]	Direct Competitive Chemiluminescent Immunoassay	Intervals set as ≥20 ng/mL (50 nmol/L), 10–19.9 ng/mL (25–49.9 nmol/L), 5–9.9 ng/mL (12.5–24.9 nmol/L), <5 ng/mL (12.5 nmol/L)
[45]	No mention	Quartiles
[36]	Radioimmunoassay prior to 2008 Liquid chromatography-tandem mass spectrometry after 2018	Intervals set as <20 ng/mL (50 nmol/L), 20–29.9 ng/mL (50–75 nmol/L), ≥30 ng/mL (75 nmol/L)
[35]	No mention	Quartiles
[46]	Isotope-dilution liquid chromatography	Intervals set as <25 nmol/L, 25–37.5 nmol/L, 37.5–50 nmol/L, 50–75 nmol/L, 75–100 nmol/L, ≥100 nmol/L
[47]	Competitive Protein Binding Assay	Intervals set as <20 ng/mL (50 nmol/L), 20–29.9 ng/mL (50–75 nmol/L), ≥30 ng/mL (75 nmol/L)
[48]	Direct Competitive Chemiluminescent Immunoassay	Intervals set as <25.0 nmol/L, 25.0–49.9 nmol/L, 50.0–74.9 nmol/L, ≥75.0 nmol/L
[49]	No mention	Intervals set as highestcategory: >65 nmol/L lowest category: <35 nmol/L

**Table 6:** Clinical trials of VD/VDA use in PC (from Pubmed indexed articles, accessed on 1 May, 2024).

Ref.	Treatment	Design	Phase	Population	No.	Results
[129]	Seocalcitol (EB1089) 20 μg orally, with dose escalation every 2 weeks until hypercalcemia occurred	Single group	II	Inoperable PC patients	36	No association between individual dose of Seocalcitol and TTF or OS.
[130]	Calcitriol 0.5 μg/kg orally over 4 h (total dose divided in 4 equal parts, administered each hour) day 1; docetaxel 36 mg/m^2^ IV over 15–30 min day 2; repeated weekly for 3 weeks with a 1 week break	Single group	II	Inoperable PDAC patients	25	Median TTP was 15 weeks and median OS was 24 weeks. A modest increase in TTP was observed when the results were compared to historical findings on docetaxel treatment only.
[131]	Cohort 1: paricalcitriol 75 mcg IV weekly + nanoliposomal irinotecan+ 5-FU/leucovorin Cohort 2: paricalcitriol 7 mcg/kg IV weekly + nanoliposomal irinotecan + 5-FU/leucovorin	Open label	Pilot	Inoperable PDAC patients that progressed/intolerant to gemcitabine chemotherapy	20	Combination therapy is safely tolerated. Correlative studies showed increased tumor vascularity in post-treatment samples in patients receiving the higher dose of paricalcitol (cohort 2).

**Table 7:** Ongoing or completed clinical trials of VD/VDA use in PDAC (from clinicaltrial.gov database, accessed on October 27th 2024).

Identifiant	Design	Phase	Target population	Treatment	Primaryoutcome	Status	Results
NCT03520790	Randomized Parallel assignment Triple-blind Placebo controlled	II	Previously untreated, metastatic PDAC	Cohort 1: gemcitabine + nab-paclitaxel + placebo Cohort 2: gemcitabine + nab-paclitaxel + paricalcitol IV Cohort 3: gemcitabine + nab-paclitaxel + paricalcitol oral	Assessing OS between treatment arms.	Active, not recruiting.	
NCT03331562	Randomized Parallel assignment Quadruple-blind Placebo controlled	III	Metastatic PDAC patients that responded to first line chemotherapy (at least SD)	Cohort 1: pembrolizumab + paricalcitol Cohort 2: pembrolizumab + placebo	Assessing PFS at 6 months between treatment arms.	Completed	No improvement in PFS.
NCT03415854	Single group Open label	II	Previously untreated, metastatic PDAC	Paclitaxelproteinbound + cisplatin + gemcitabine Addition Of Paricalcitol in case of diseaseprogression	Assessing the reponse rate at the end of 3 treatment cycles.	Completed	Data not available.
NCT02030860	Randomized Open label	I	Previously untreated, apparently resectable PDAC	Cohort 1: preoperativegemcitabine + abraxane + paricalcitol Cohort 2: preoperativegemcitabine + abraxane	Assessing the adverse effects of treatments.	Completed	Data not available.
NCT04617067	Single group Open label	II	Advanced PDAC	Gemcitabine + nab-paclitaxel + paricalcitol	Assessing PFS at 24 weeks.	Completed	Data not available.
NCT03138720	Single group Open label	II	Non-metastatic PDAC.	Paclitaxelproteinbound + gemcitabine + cisplatin + paricalcitol	Assessing CA19-9 evolution after 3 cycles of treatment	Active, not recruiting.	
NCT04524702	Single group Open label	II	Advanced PDAC	Paricalcitol+ hydroxychloroquine + gemcitabine + nab-paclitaxel	Assessing change in tumor size at 8 weeks of treatment.	Active, not recruiting.	
NCT05365893	Non-randomized Open label	I	Resecable PDAC	Cohort 1: neoadjuvanttherapywithmodified FOLFIRINOX andchemoradiation + neodjuvantparicalcitol, hydroxychloroquine, andlosartan + surgery Cohort 2: neoadjuvanttherapywithmodified FOLFIRINOX andchemoradiation + surgery	Assessing the adverse effects of treatments.	Recruiting	
NCT02930902	Non-randomized Open label	I	Resecable PDAC	Cohort 1: pembrolizumab + paricalcitol Cohort 2: pembrolizumab + paricalcitol + gemcitabine + nab-paclitaxel	Assessing the adverse effects of treatments.	Completed	Data not available.
NCT02754726	Single group Open label	II	Untreated metastatic	Nivolumab + albumin-boundpaclitaxel + Paricalcitol + cisplatin + gemcitabine	Assessing the reponse rate of the treatment.	Active, not recruiting.	

## Data Availability

Not applicable.

## Abbreviations

VD	vitamin D
VDR	vitamin D receptor
VDA	vitamin D analog/superagonist
VDB	vitamin D binding receptor
VDRE	vitamin D response element
RXR	retinoid X Receptor
CYP2R1	25(OH)-hydroxylase
CYP27B1	1α-hydroxylase
CY24A1	24(OH)-hydroxylase
SNP	single nucleotide polymorphism
PC	pancreatic cancer
PDAC	pancreatic ductal adenocarcinoma
PanIN	pancreatic intraepithelial neoplasia
CRT	chemoradiotherapy
EMT	epithelial-to-mesenchymal transition
TME	tumor microenvironment
PSC	pancreatic stellate cell
CAF	cancer associated fibroblast

## References

[1] Aranow C. (2011). Vitamin D and the Immune System. J. Investig. Med..

[2] Samuel S., Sitrin M.D. (2008). Vitamin D’s Role in Cell Proliferation and Differentiation. Nutr. Rev..

[3] Peller S., Stephenson C. (1937). Skin Irritation and Cancer in the United States Navy. Am. J. Med. Sci. Am. J. Med. Sci..

[4] Apperly F. (1941). The Relation of Solar Radiation to Cancer Mortality in North American. Cancer Res..

[5] American Cancer Society (2024). Cancer Facts & Figures 2024.

[6] Holick M., McLaughlin J., Holick S., Potts J.T., Anderson R.R., Blank I.H., Parrish J.A., Elias P. (1980). Photosynthesis of Previtamin D3 in Human and the Physiologic Consequences. Science (1979).

[7] Webb A., DeCosta B., Holick M. (1989). Sunlight Regulates the Cutaneous Production of Vitamin D3 by Causing Its Photodegradation. J. Clin. Endocrinol. Metab..

[8] Tripkovic L., Lambert H., Hart K., Smith C.P., Bucca G., Penson S., Chope G., Hyppönen E., Berry J., Vieth R. (2012). Comparison of Vitamin D2 and Vitamin D3 Supplementation in Raising Serum 25-Hydroxyvitamin D Status: A Systematic Review and Meta-Analysis. Am. J. Clin. Nutr..

[9] Sugimoto H., Shiro Y. (2012). Diversity and Substrate Specificity in the Structures of Steroidogenic Cytochrome P450 Enzymes. Biol. Pharm. Bull..

[10] Zierold C., Reinholz G.G., Mings J.A., Prahl J.M., DeLuca H.F. (2000). Regulation of the Porcine 1,25-Dihydroxyvitamin D3-24-Hydroxylase (CYP24) by 1,25-Dihydroxyvitamin D3 and Parathyroid Hormone in AOK-B50 Cells. Arch. Biochem. Biophys..

[11] Zehnder D., Bland R., Williams M.C., McNinch R.W., Howie A.J., Stewart P.M., Hewison M. (2001). Extrarenal Expression of 25-Hydroxyvitamin D_3_ -1α-Hydroxylase ^1^. J. Clin. Endocrinol. Metab..

[12] Stumpf W.E., Sar M., Reid F.A., Tanaka Y., DeLuca H.F. (1979). Target Cells for 1,25-Dihydroxyvitamin D_3_ in Intestinal Tract, Stomach, Kidney, Skin, Pituitary, and Parathyroid. Science (1979).

[13] Anderson P.H., O’Loughlin P.D., May B.K., Morris H.A. (2005). Modulation of CYP27B1 and CYP24 MRNA Expression in Bone Is Independent of Circulating 1,25(OH)2D3 Levels. Bone.

[14] Young M.V. (2004). The Prostate 25-Hydroxyvitamin D-1 -Hydroxylase Is Not Influenced by Parathyroid Hormone and Calcium: Implications for Prostate Cancer Chemoprevention by Vitamin D. Carcinogenesis.

[15] Piper M.R., Freedman D.M., Robien K., Kopp W., Rager H., Horst R.L., Stolzenberg-Solomon R.Z. (2015). Vitamin D–Binding Protein and Pancreatic Cancer: A Nested Case-Control Study. Am. J. Clin. Nutr..

[16] Nykjaer A., Dragun D., Walther D., Vorum H., Jacobsen C., Herz J., Melsen F., Christensen E.I., Willnow T.E. (1999). An Endocytic Pathway Essential for Renal Uptake and Activation of the Steroid 25-(OH) Vitamin D3. Cell.

[17] Rachez C., Freedman L.P. (2000). Mechanisms of Gene Regulation by Vitamin D3 Receptor: A Network of Coactivator Interactions. Gene.

[18] Nemere I., Farach-Carson M.C., Rohe B., Sterling T.M., Norman A.W., Boyan B.D., Safford S.E. (2004). Ribozyme Knockdown Functionally Links a 1,25(OH)_2_ D_3_ Membrane Binding Protein (1,25D_3_ -MARRS) and Phosphate Uptake in Intestinal Cells. Proc. Natl. Acad. Sci. USA.

[19] Mizwicki M.T., Norman A.W. (2009). The Vitamin D Sterol–Vitamin D Receptor Ensemble Model Offers Unique Insights into Both Genomic and Rapid-Response Signaling. Sci. Signal..

[20] Garland C., Garland F. (1980). Do Sunlight and Vitamin D Reduce the Likelihood of Colon Cancer?. Int. J. Epidemiol..

[21] Garland F., Garland C., Gorham E. (1990). Geographic Variation in Breast Cancer Mortality in the United States: A Hypothesis Involving Exposure to Solar Radiation. Prev. Med..

[22] Lefkowitz E., Garland F. (1994). Sunlight, Vitamin D and Ovarian Cancer Mortality Rates in US Women. Int. J. Epidemiol..

[23] Hanchette C., Schwartz G. (1992). Geographic Patterns of Prostate Cancer Mortality. Cancer.

[24] Boscoe F.P., Schymura M.J. (2006). Solar Ultraviolet-B Exposure and Cancer Incidence and Mortality in the United States, 1993–2002. BMC Cancer.

[25] Mohr S.B., Garland C.F., Gorham E.D., Grant W.B., Garland F.C. (2010). Ultraviolet B Irradiance and Vitamin D Status Are Inversely Associated With Incidence Rates of Pancreatic Cancer Worldwide. Pancreas.

[26] Lindelöf B., Krynitz B., Ayoubi S., Martschin C., Wiegleb-Edström D., Wiklund K. (2012). Previous Extensive Sun Exposure and Subsequent Vitamin D Production in Patients with Basal Cell Carcinoma of the Skin, Has No Protective Effect on Internal Cancers. Eur. J. Cancer.

[27] Skinner H.G., Michaud D.S., Giovannucci E., Willett W.C., Colditz G.A., Fuchs C.S. (2006). Vitamin D Intake and the Risk for Pancreatic Cancer in Two Cohort Studies. Cancer Epidemiol. Biomark. Prev..

[28] Zablotska L.B., Gong Z., Wang F., Holly E.A., Bracci P.M. (2011). Vitamin D, Calcium, and Retinol Intake, and Pancreatic Cancer in a Population-Based Case-Control Study in the San Francisco Bay Area. Cancer Causes Control.

[29] Waterhouse C.M., Risch H.A., Bosetti C., Anderson K.E., Petersen G.M., Bamlet W.R., Cotterchio M., Cleary S.P., Ibiebele T., Vecchia C.L. (2015). Vitamin D and Pancreatic Cancer: A Pooled Analysis from the Pancreatic Cancer Case-Control consortium. Ann. Oncol..

[30] Stolzenberg-Solomon R.Z., Pietinen P., Taylor P.R., Virtamo J., Albanes D. (2002). Prospective Study of Diet and Pancreatic Cancer in Male Smokers. Am. J. Epidemiol. Orig. Contrib..

[31] Baghurst P.A., Mcmichael A.J., Slavotinek A.H., Baghurst K.I., Boyle P., Walker A.M. (1991). A Case-Control Study of Diet and Cancer of the Pancreas. Am. J. Epidemiol..

[32] Bravi F., Polesel J., Bosetti C., Talamini R., Negri E., Dal Maso L., Serraino D., La Vecchia C. (2011). Dietary Intake of Selected Micronutrients and the Risk of Pancreatic Cancer: An Italian Case-Control Study. Ann. Oncol..

[33] Jansen R.J., Robinson D.P., Frank R.D., Anderson K.E., Bamlet W.R., Oberg A.L., Rabe K.G., Olson J.E., Sinha R., Petersen G.M. (2014). Fatty Acids Found in Dairy, Protein and Unsaturated Fatty Acids Are Associated with Risk of Pancreatic Cancer in a Case-Control Study. Int. J. Cancer.

[34] Genkinger J.M., Wang M., Li R., Albanes D., Anderson K.E., Bernstein L., van den Brandt P.A., English D.R., Freudenheim J.L., Fuchs C.S. (2014). Dairy Products and Pancreatic Cancer Risk: A Pooled Analysis of 14 Cohort Studies. Ann. Oncol..

[35] Zhang X., Huang X.-Z., Chen W.-J., Wu J., Chen Y., Wu C.-C., Wang Z.-N. (2017). Plasma 25-Hydroxyvitamin D Levels, Vitamin D Intake, and Pancreatic Cancer Risk or Mortality: A Meta-Analysis. Oncotarget.

[36] Ananthakrishnan A.N., Cheng S.C., Cai T., Cagan A., Gainer V.S., Szolovits P., Shaw S.Y., Churchill S., Karlson E.W., Murphy S.N. (2014). Association between Reduced Plasma 25-Hydroxy Vitamin D and Increased Risk of Cancer in Patients with Inflammatory Bowel Diseases. Clin. Gastroenterol. Hepatol..

[37] Afzal S., Bojesen S.E., Nordestgaard B.G. (2013). Low Plasma 25-Hydroxyvitamin D and Risk of Tobacco-Related Cancer. Clin. Chem..

[38] Stolzenberg-Solomon R.Z., Hayes R.B., Horst R.L., Anderson K.E., Hollis B.W., Silverman D.T. (2009). Serum Vitamin D and Risk of Pancreatic Cancer in the Prostate, Lung, Colorectal, and Ovarian Screening Trial. Cancer Res..

[39] Wolpin B.M., Ng K., Bao Y., Kraft P., Stampfer M.J., Michaud D.S., Ma J., Buring J.E., Sesso H.D., Lee I.M. (2012). Plasma 25-Hydroxyvitamin D and Risk of Pancreatic Cancer. Cancer Epidemiol. Biomark. Prev..

[40] Liu Y., Wang X., Sun X., Lu S., Liu S. (2018). Vitamin Intake and Pancreatic Cancer Risk Reduction. Medicine.

[41] Stolzenberg-Solomon R.Z., Vieth R., Azad A., Pietinen P., Taylor P.R., Virtamo J., Albanes D. (2006). A Prospective Nested Case-Control Study of Vitamin D Status and Pancreatic Cancer Risk in Male Smokers. Cancer Res..

[42] Bao Y., Ng K., Wolpin B.M., Michaud D.S., Giovannucci E., Fuchs C.S. (2010). Predicted Vitamin D Status and Pancreatic Cancer Risk in Two Prospective Cohort Studies. Br. J. Cancer.

[43] Stolzenberg-Solomon R.Z., Jacobs E.J., Arslan A.A., Qi D., Patel A.V., Helzlsouer K.J., Weinstein S.J., McCullough M.L., Purdue M.P., Shu X.O. (2010). Circulating 25-Hydroxyvitamin D and Risk of Pancreatic Cancer: Cohort Consortium Vitamin D Pooling Project of Rarer Cancers. Am. J. Epidemiol..

[44] Weinstein S.J., Stolzenberg-Solomon R.Z., Kopp W., Rager H., Virtamo J., Albanes D. (2012). Impact of Circulating Vitamin D Binding Protein Levels on the Association between 25-Hydroxyvitamin d and Pancreatic Cancer Risk: A Nested Case-Control Study. Cancer Res..

[45] Liu S.L., Zhao Y.P., Dai M.H., You L., Wen Z., Xu J.W. (2013). Vitamin D Status and the Risk of Pancreatic Cancer: A Meta-Analysis. Chin. Med. J..

[46] van Duijnhoven F.J.B., Jenab M., Hveem K., Siersema P.D., Fedirko V., Duell E.J., Kampman E., Halfweeg A., van Kranen H.J., van den Ouweland J.M.W. (2018). Circulating Concentrations of Vitamin D in Relation to Pancreatic Cancer Risk in European Populations. Int. J. Cancer.

[47] Mohamed A., Aref A., Talima S., A Elshimy R., Gerges S., Meghed M., Zahran F., El-Adawy E., Abd-Elsalam S. (2019). Association of Serum Level of Vitamin D and VDR Polymorphism Fok1 with the Risk or Survival of Pancreatic Cancer in Egyptian Population. Indian. J. Cancer.

[48] Wu E., Guo J.P., Wang K., Xu H.Q., Xie T., Tao L., Ni J.T. (2023). Association of Serum 25-Hydroxyvitamin D with the Incidence of 16 Cancers, Cancer Mortality, and All-Cause Mortality among Individuals with Metabolic Syndrome: A Prospective Cohort Study. Eur. J. Nutr..

[49] Shen Y., Xia J., Yi C., Li T., Wang P., Dai L., Shi J., Wang K., Sun C., Ye H. (2024). The Association between Circulating 25-Hydroxyvitamin D and Pancreatic Cancer: A Systematic Review and Meta-Analysis of Observational Studies. Eur. J. Nutr..

[50] Giovannucci E., Liu Y., Rimm E.B., Hollis B.W., Fuchs C.S., Stampfer M.J., Willett W.C. (2006). Prospective Study of Predictors of Vitamin D Status and Cancer Incidence and Mortality in Men. J. Natl. Cancer Inst..

[51] Li L., Wu B., Yang L., Yin G., Wei W., Sui S., Liu J. (2013). Association of Vitamin D Receptor Gene Polymorphisms with Pancreatic Cancer: A Pilot Study in a North China Population. Oncol. Lett..

[52] Anderson L.N., Cotterchio M., Knight J.A., Borgida A., Gallinger S., Cleary S.P. (2013). Genetic Variants in Vitamin D Pathway Genes and Risk of Pancreas Cancer; Results from a Population-Based Case-Control Study in Ontario, Canada. PLoS ONE.

[53] Arem H., Yu K., Xiong X., Moy K., Freedman N.D., Mayne S.T., Albanes D., Arslan A.A., Austin M., Bamlet W.R. (2015). Vitamin D Metabolic Pathway Genes and Pancreatic Cancer Risk. PLoS ONE.

[54] Li L., Shang F., Zhang W., Zhang C., Li J., Wang C., Wei L. (2015). Role of Vitamin D Receptor Gene Polymorphisms in Pancreatic Cancer: A Case–Control Study in China. Tumor Biol..

[55] Zhu M., Qiu S., Zhang X., Wang Y., Souraka T.D.M., Wen X., Liang C., Tu J. (2018). The Associations between CYP24A1 Polymorphisms and Cancer Susceptibility: A Meta-Analysis and Trial Sequential Analysis. Pathol. Res. Pract..

[56] Dimitrakopoulou V.I., Tsilidis K.K., Haycock P.C., Dimou N.L., Al-Dabhani K., Martin R.M., Lewis S.J., Gunter M.J., Mondul A., Shui I.M. (2017). Circulating Vitamin D Concentration and Risk of Seven Cancers: Mendelian Randomisation Study. BMJ.

[57] Ong J.S., Gharahkhani P., An J., Law M.H., Whiteman D.C., Neale R.E., MacGregor S. (2018). Vitamin D and Overall Cancer Risk and Cancer Mortality: A Mendelian Randomization Study. Hum. Mol. Genet..

[58] Chen B., Diallo M.T., Ma Y., Wang D. (2023). The Association of Vitamin D and Digestive System Cancers: A Comprehensive Mendelian Randomization Study. J. Cancer Res. Clin. Oncol..

[59] Weinstein S.J., Mondul A.M., Yu K., Layne T.M., Abnet C.C., Freedman N.D., Stolzenberg-Solomon R.Z., Lim U., Gail M.H., Albanes D. (2018). Circulating 25-Hydroxyvitamin D up to 3 Decades Prior to Diagnosis in Relation to Overall and Organ-Specific Cancer Survival. Eur. J. Epidemiol..

[60] Jiang X., Kiel D.P., Kraft P. (2019). The Genetics of Vitamin D. Bone.

[61] Wolski H., Kurzawińska G., Ożarowski M., Mrozikiewicz A.E., Drews K., Karpiński T.M., Bogacz A., Seremak-Mrozikiewicz A. (2021). Vitamin D Receptor Gene Polymorphisms and Haplotypes in the Etiology of Recurrent Miscarriages. Sci. Rep..

[62] Grant W.B. (2002). An Estimate of Premature Cancer Mortality in the U.S. Due to Inadequate Doses of Solar Ultraviolet-B Radiation. Cancer.

[63] Grant W.B. (2002). An Ecologic Study of the Role of Solar UV-B Radiation in Reducing the Risk of Cancer Using Cancer Mortality Data, Dietary Supply Data, and Latitude for European Countries. Biologic Effects of Light 2001: Proceedings of a Symposium Boston, Massachusetts, Boston, MA, USA, 16–18 June 2001.

[64] Grant W.B., Grant W.B., Place F.W. (2003). Ecologic Studies of Solar UV-B Radiation and Cancer Mortality Rates. Vitam. D Analog. Cancer Prev. Ther..

[65] Mizoue T. (2004). Ecological Study of Solar Radiation and Cancer Mortality in Japan. Health Phys..

[66] Kinoshita S., Wagatsuma Y., Okada M. (2007). Geographical Distribution for Malignant Neoplasm of the Pancreas in Relation to Selected Climatic Factors in Japan. Int. J. Health Geogr..

[67] Grant W.B. (2007). An Ecologic Study of Cancer Mortality Rates in Spain with Respect to Indices of Solar UVB Irradiance and Smoking. Int. J. Cancer.

[68] Neale R.E., Youlden D.R., Krnjacki L., Kimlin M.G., Van Der Pols J.C. (2009). Latitude Variation in Pancreatic Cancer Mortality in Australia. Pancreas.

[69] Haas M., Kern C., Kruger S., Michl M., Modest D.P., Giessen C., Schulz C., von Einem J.C., Ormanns S., Laubender R.P. (2015). Assessing Novel Prognostic Serum Biomarkers in Advanced Pancreatic Cancer: The Role of CYFRA 21-1, Serum Amyloid A, Haptoglobin, and 25-OH Vitamin D3. Tumor Biol..

[70] McGovern E.M., Lewis M.E., Niesley M.L., Huynh N., Hoag J.B. (2016). Retrospective Analysis of the Influence of 25-Hydroxyvitamin D on Disease Progression and Survival in Pancreatic Cancer. Nutr. J..

[71] Von Hoff D.D., Cridebring D., Tian O.Y., Han H., Bhore R., Franco T., Ondovik M.S., Louis C.U. (2021). Analysis of the Role of Plasma 25-Hydroxyvitamin D Levels in Survival Outcomes in Patients from the Phase III MPACT Trial of Metastatic Pancreatic Cancer. Oncologist.

[72] Van Loon K., Owzar K., Jiang C., Kindler H.L., Mulcahy M.F., Niedzwiecki D., O’Reilly E.M., Fuchs C., Innocenti F., Venook A.P. (2014). 25-Hydroxyvitamin D Levels and Survival in Advanced Pancreatic Cancer: Findings from CALGB 80303 (Alliance). J. Natl. Cancer Inst..

[73] Cho M., Peddi P.F., Ding K., Chen L., Thomas D., Wang J., Lockhart A.C., Tan B., Wang-Gillam A. (2013). Vitamin D Deficiency and Prognostics among Patients with Pancreatic Adenocarcinoma. J. Transl. Med..

[74] Yuan C., Qian Z.R., Babic A., Morales-Oyarvide V., Rubinson D.A., Kraft P., Ng K., Bao Y., Giovannucci E.L., Ogino S. (2016). Prediagnostic Plasma 25-Hydroxyvitamin D and Pancreatic Cancer Survival. J. Clin. Oncol..

[75] Rasmussen L.S., Yilmaz M.K., Falkmer U.G., Poulsen L., Bøgsted M., Christensen H.S., Bojesen S.E., Jensen B.V., Chen I.M., Johansen A.Z. (2021). Pre-Treatment Serum Vitamin D Deficiency Is Associated with Increased Inflammatory Biomarkers and Short Overall Survival in Patients with Pancreatic Cancer. Eur. J. Cancer.

[76] Mohammed A.A., Salem R.A. (2021). Impact of Vitamin D Serum Levels on Clinicopathological Features and Outcome in Advanced Pancreatic Carcinoma. Forum Clin. Oncol..

[77] Li J., Zhang H., Zhu H., Dai Z. (2023). 25-Hydroxyvitamin D Concentration Is Positively Associated with Overall Survival in Advanced Pancreatic Cancer: A Systematic Review and Meta-Analysis. Nutr. Res..

[78] Mukai Y., Yamada D., Eguchi H., Iwagami Y., Asaoka T., Noda T., Kawamoto K., Gotoh K., Kobayashi S., Takeda Y. (2018). Vitamin D Supplementation Is a Promising Therapy for Pancreatic Ductal Adenocarcinoma in Conjunction with Current Chemoradiation Therapy. Ann. Surg. Oncol..

[79] Wang K., Dong M., Sheng W., Liu Q., Yu D., Dong Q., Li Q., Wang J. (2015). Expression of Vitamin D Receptor as a Potential Prognostic Factor and Therapeutic Target in Pancreatic Cancer. Histopathology.

[80] Bahat A.V., Bar-David S., Brooks A., Aizic A., Greenberg O., Wolf I., Klausner J.M., Lahat G., Nizri E. (2020). Protective Desmoplasia in Pancreatic Adenocarcinoma: High Vitamin D Receptor Expression and Collagen Content. Anticancer. Res..

[81] Li H., Ruan Y., Liu C., Fan X., Yao Y., Dai Y., Song Y., Jiang D., Sun N., Jiao G. (2024). VDR Promotes Pancreatic Cancer Progression in Vivo by Activating CCL20-Mediated M2 Polarization of Tumor Associated Macrophage. Cell Commun. Signal..

[82] Gao W., Wang T.F., Yi W., Tong W.M. (2020). The Effects of CYP24A1 on Clinicopathological Features and the Prognosis of Pancreatic Ductal Adenocarcinoma. Transl. Cancer Res..

[83] Innocenti F., Owzar K., Jiang C., Etheridge A.S., Gordân R., Sibley A.B., Mulkey F., Niedzwiecki D., Glubb D., Neel N. (2018). The Vitamin D Receptor Gene as a Determinant of Survival in Pancreatic Cancer Patients: Genomic Analysis and Experimental Validation. PLoS ONE.

[84] Palamaris K., Felekouras E., Sakellariou S. (2021). Epithelial to Mesenchymal Transition: Key Regulator of Pancreatic Ductal Adenocarcinoma Progression and Chemoresistance. Cancers.

[85] Opitz F.V., Haeberle L., Daum A., Esposito I. (2021). Tumor Microenvironment in Pancreatic Intraepithelial Neoplasia. Cancers.

[86] Stefanoudakis D., Frountzas M., Schizas D., Michalopoulos N.V., Drakaki A., Toutouzas K.G. (2024). Significance of TP53, CDKN2A, SMAD4 and KRAS in Pancreatic Cancer. Curr. Issues Mol. Biol..

[87] Zhang N., Wei P., Gong A., Chiu W.-T., Lee H.-T., Colman H., Huang H., Xue J., Liu M., Wang Y. (2011). FoxM1 Promotes β-Catenin Nuclear Localization and Controls Wnt Target-Gene Expression and Glioma Tumorigenesis. Cancer Cell.

[88] Xue W., Yang L., Chen C., Ashrafizadeh M., Tian Y., Sun R. (2024). Wnt/β-Catenin-Driven EMT Regulation in Human Cancers. Cell. Mol. Life Sci..

[89] Chiang K.C., Yeh C.N., Hsu J.T., Jan Y.Y., Chen L.W., Kuo S.F., Takano M., Kittaka A., Chen T.C., Chen W.T. (2014). The Vitamin D Analog, MART-10, Represses Metastasis Potential via Downregulation of Epithelial-Mesenchymal Transition in Pancreatic Cancer Cells. Cancer Lett..

[90] Murphy K.J., Chambers C.R., Herrmann D., Timpson P., Pereira B.A. (2021). Dynamic Stromal Alterations Influence Tumor-Stroma Crosstalk to Promote Pancreatic Cancer and Treatment Resistance. Cancers.

[91] Chakkera M., Foote J.B., Farran B., Nagaraju G.P. (2024). Breaking the Stromal Barrier in Pancreatic Cancer: Advances and Challenges. Biochim. Et Biophys. Acta (BBA)-Rev. Cancer.

[92] Zhang T., Ren Y., Yang P., Wang J., Zhou H. (2022). Cancer-Associated Fibroblasts in Pancreatic Ductal Adenocarcinoma. Cell Death Dis..

[93] Saúde-Conde R., Arçay Öztürk A., Stosic K., Azurmendi Senar O., Navez J., Bouchart C., Arsenijevic T., Flamen P., Van Laethem J.-L. (2024). Cancer-Associated Fibroblasts in Pancreatic Ductal Adenocarcinoma or a Metaphor for Heterogeneity: From Single-Cell Analysis to Whole-Body Imaging. Biomedicines.

[94] Rhim A.D., Oberstein P.E., Thomas D.H., Mirek E.T., Palermo C.F., Sastra S.A., Dekleva E.N., Saunders T., Becerra C.P., Tattersall I.W. (2014). Stromal Elements Act to Restrain, Rather Than Support, Pancreatic Ductal Adenocarcinoma. Cancer Cell.

[95] Colston K.W., James’ S.Y., Ofori-Kuragul E.A., Binderup L., Grant A.G. (1997). Vitamin D Receptors and Anti-Proliferative Effects of Vitamin D Derivatives in Human Pancreatic Carcinoma Cells in Vivo and in Vitro. Br. J. Cancer.

[96] Schwartz G.G., Eads D., Rao A., Cramer S.D., Willingham M.C., Chen T.C., Jamieson D.P., Wang L., Burnstein K.L., Holick M.F. (2004). Pancreatic Cancer Cells Express 25-Hydroxyvitamin D-1α-Hydroxylase and Their Proliferation Is Inhibited by the Prohormone 25-Hydroxyvitamin D3. Carcinogenesis.

[97] Albrechtsson E., Jonsson T., Möller S., Höglund M., Ohlsson B., Axelson J. (2003). Vitamin D Receptor Is Expressed in Pancreatic Cancer Cells and a Vitamin D3 Analogue Decreases Cell Number. Pancreatology.

[98] Kawal S., Nikaido T., Aoki’ Y., Zhai Y., Kumagai T., Furihata K., Fujii S., Kiyosawa K. (1997). Vitamin D Analogues Upmregulate P21 and P27 during Growth Inhibition of Pancreatic Cancer Cell Lines. Br. J. Cancer.

[99] Kawa S., Yoshizawa K., Nikaido T., Kiyosawa K. (2005). Inhibitory Effect of 22-Oxa-1,25-Dihydroxyvitamin D3, Maxacalcitol, on the Proliferation of Pancreatic Cancer Cell Lines. J. Steroid Biochem. Mol. Biol..

[100] Schwartz G.G., Eads D., Naczki C., Northrup S., Chen T., Koumenis C. (2008). 19-nor-1α,25-Dihydroxyvitamin D2 (Paricalcitol) Inhibits the Proliferation of Human Pancreatic Cancer Cells in Vitro and in Vivo. Cancer Biol. Ther..

[101] Chiang K.C., Yeh C.N., Hsu J.T., Yeh T.S., Jan Y.Y., Wu C.T., Chen H.Y., Jwo S.C., Takano M., Kittaka A. (2013). Evaluation of the Potential Therapeutic Role of a New Generation of Vitamin D Analog, MART-10, in Human Pancreatic Cancer Cells in Vitro and in Vivo. Cell Cycle.

[102] Kanemaru M., Maehara N., Chijiwa K. (2013). Antiproliferative Effect of 1α,25-Dihydroxyvitamin D3 Involves Upregulation of Cyclin-Dependent Kinase Inhibitor P21 in Human Pancreatic Cancer Cells. Hepatogastroeneterology.

[103] Brüggemann L.W., Queiroz K.C.S., Zamani K., Van Straaten A., Spek C.A., Bijlsma M.F. (2010). Assessing the Efficacy of the Hedgehog Pathway Inhibitor Vitamin D3 in a Murine Xenograft Model for Pancreatic Cancer. Cancer Biol. Ther..

[104] Yu W.D., Ma Y., Flynn G., Muindi J.R., Kong R.X., Trump D.L., Johnson C.S. (2010). Calcitriol Enhances Gemcitabine Anti-Tumor Activity in Vitro and in Vivo by Promoting Apoptosis in a Human Pancreatic Carcinoma Model System. Cell Cycle.

[105] Persons K.S., Eddy V.J., Chadid S., Deoliveira R., Saha A.K., Ray R. (2010). Anti-Growth Effect of 1,25-Dihydroxyvitamin D 3-3-Bromoacetate Alone or in Combination with 5-Amino-Imidazole-4-Carboxamide-1-β-4-Ribofuranoside in Pancreatic Cancer Cells. Anticancer. Res..

[106] Arensman M.D., Nguyen P., Kershaw K.M., Lay A.R., Ostertag-Hill C.A., Sherman M.H., Downes M., Liddle C., Evans R.M., Dawson D.W. (2015). Calcipotriol Targets LRP6 to Inhibit Wnt Signaling in Pancreatic Cancer. Mol. Cancer Res..

[107] Li Z., Jia Z., Gao Y., Xie D., Wei D., Cui J., Mishra L., Huang S., Zhang Y., Xie K. (2015). Activation of Vitamin D Receptor Signaling Downregulates the Expression of Nuclear FOXM1 Protein and Suppresses Pancreatic Cancer Cell Stemness. Clin. Cancer Res..

[108] He B., Stoffel L., He C.J., Cho K., Li A.M., Jiang H., Flowers B.M., Nguyen K.T., Wang K.W., Zhao A.Y. (2024). Epigenetic Priming Targets Tumor Heterogeneity to Shift Transcriptomic Phenotype of Pancreatic Ductal Adenocarcinoma towards a Vitamin D Susceptible State. Cell Death Dis..

[109] Pettersson F., Colston K.W., Dalgleish A.G. (2000). Differential and Antagonistic Effects of 9-Cis-Retinoic Acid and Vitamin D Analogues on Pancreatic Cancer Cells in Vitro. Br. J. Cancer.

[110] Mouratidis P.X.E., Dalgleish A.G., Colston K.W. (2006). Investigation of the Mechanisms by Which EB1089 Abrogates Apoptosis Induced by 9-Cis Retinoic Acid in Pancreatic Cancer Cells. Pancreas.

[111] Kawa S., Yoshizawa K., Tokoo M., Imai H., Oguchi H., Kiyosawa K., Homma T., Nikaido T., Furihata K. (1996). Inhibitory Effect of 22-Oxa-1,25-Dihydroxyvitamin D 3 on the Proliferation of Pancreatic Cancer Cell Lines. Gastroenterology.

[112] Zugmaierl G., Jigerl R., Grage B., Gottardis M.M., Havemann K., Knabbe C. (1996). Growth-Inhibitory Effects of Vitamin D Analogues and Retinoids on Human Pancreatic Cancer Cells. Br. J. Cancer.

[113] Ohlsson B., Albrechtsson E., Axelson J. (2004). Vitamins A and D but Not E and K Decreased the Cell Number in Human Pancreatic Cancer Cell Lines. Scand. J. Gastroenterol..

[114] Ramalho M.J., Loureiro J.A., Gomes B., Frasco M.F., Coelho M.A.N., Carmo Pereira M. (2015). PLGA Nanoparticles as a Platform for Vitamin D-Based Cancer Therapy. Beilstein J. Nanotechnol..

[115] Dooley J., Lagou V., Heirman N., Dresselaers T., Himmelreich U., Liston A. (2017). Murine Pancreatic Acinar Cell Carcinoma Growth Kinetics Are Independent of Dietary Vitamin D Deficiency or Supplementation. Front. Oncol..

[116] Sherman M.H., Yu R.T., Engle D.D., Ding N., Atkins A.R., Tiriac H., Collisson E.A., Connor F., Van Dyke T., Kozlov S. (2014). Vitamin D Receptor-Mediated Stromal Reprogramming Suppresses Pancreatitis and Enhances Pancreatic Cancer Therapy. Cell.

[117] Bhattacharjee V., Zhou Y., Yen T.J. (2014). A Synthetic Lethal Screen Identifies the Vitamin D Receptor as a Novel Gemcitabine Sensitizer in Pancreatic Cancer Cells. Cell Cycle.

[118] Li L., Shang F., Zhu Y., Sun Y., Sudi R.S. (2019). Modulation of VDR and Cell Cycle-Related Proteins by Vitamin D in Normal Pancreatic Cells and Poorly Differentiated Metastatic Pancreatic Cancer Cells. Nutr. Cancer.

[119] Kong F., Li L., Wang G., Deng X., Li Z., Kong X. (2019). VDR Signaling Inhibits Cancer-Associated-Fibroblasts’ Release of Exosomal MiR-10a-5p and Limits Their Supportive Effects on Pancreatic Cancer Cells. Gut.

[120] Porter R.L., Magnus N.K.C., Thapar V., Morris R., Szabolcs A., Neyaz A., Kulkarni A.S., Tai E., Chougule A., Hillis A. (2019). Epithelial to Mesenchymal Plasticity and Differential Response to Therapies in Pancreatic Ductal Adenocarcinoma. Proc. Natl. Acad. Sci. USA.

[121] Moz S., Contran N., Facco M., Trimarco V., Plebani M., Basso D. (2020). Vitamin D Prevents Pancreatic Cancer-Induced Apoptosis Signaling of Inflammatory Cells. Biomolecules.

[122] Gorchs L., Ahmed S., Mayer C., Knauf A., Fernández Moro C., Svensson M., Heuchel R., Rangelova E., Bergman P., Kaipe H. (2020). The Vitamin D Analogue Calcipotriol Promotes an Anti-Tumorigenic Phenotype of Human Pancreatic CAFs but Reduces T Cell Mediated Immunity. Sci. Rep..

[123] Kang Z., Wang C., Tong Y., Li Y., Gao Y., Hou S., Hao M., Han X., Wang B., Wang Q. (2021). Novel Nonsecosteroidal Vitamin D Receptor Modulator Combined with Gemcitabine Enhances Pancreatic Cancer Therapy through Remodeling of the Tumor Microenvironment. J. Med. Chem..

[124] Cheng Y.H., Chiang E.P.I., Syu J.N., Chao C.Y., Lin H.Y., Lin C.C., Yang M.D., Tsai S.Y., Tang F.Y. (2021). Treatment of 13-Cis Retinoic Acid and 1,25- Dihydroxyvitamin D3 Inhibits TNF-Alphamediated Expression of MMP-9 Protein and Cell Invasion through the Suppression of JNK Pathway and MicroRNA 221 in Human Pancreatic Adenocarcinoma Cancer Cells. PLoS ONE.

[125] Fiz C., Apprato G., Ricca C., Aillon A., Bergandi L., Silvagno F. (2021). Tgf Beta Induces Vitamin d Receptor and Modulates Mitochondrial Activity of Human Pancreatic Cancer Cells. Cancers.

[126] Kim S.I., Chaurasiya S., Sivanandam V., Kang S., Park A.K., Lu J., Yang A., Zhang Z., Bagdasarian I.A., Woo Y. (2022). Priming Stroma with a Vitamin D Analog to Optimize Viroimmunotherapy for Pancreatic Cancer. Mol. Ther. Oncolytics.

[127] Kong W., Liu Z., Sun M., Liu H., Kong C., Ma J., Wang R., Qian F. (2022). Synergistic Autophagy Blockade and VDR Signaling Activation Enhance Stellate Cell Reprogramming in Pancreatic Ductal Adenocarcinoma. Cancer Lett..

[128] Wei D., Wang L., Liu Y., Hafley M.A., Tan L., Lorenzi P.L., Yang P., Zuo X., Bresalier R.S. (2023). Activation of Vitamin D/VDR Signaling Reverses Gemcitabine Resistance of Pancreatic Cancer Cells Through Inhibition of MUC1 Expression. Dig. Dis. Sci..

[129] Evans T., Colston K.W., Lofts F.J., Cunningham D., Anthoney D.A., Gogas H., De Bono J.S., Hamberg K.J., Skov T., Mansi J.L. (2002). A Phase II Trial of the Vitamin D Analogue Seocalcitol (EB1089) in Patients with Inoperable Pancreatic Cancer. Br. J. Cancer.

[130] Blanke C.D., Beer T.M., Todd K., Mori M., Stone M., Lopez C. (2009). Phase II Study of Calcitriol-Enhanced Docetaxel in Patients with Previously Untreated Metastatic or Locally Advanced Pancreatic Cancer. Investig. New Drugs.

[131] Grierson P.M., Suresh R., Tan B., Pedersen K.S., Amin M., Park H., Trikalinos N.A., Liu J., Boice N., Brown A. (2023). A Pilot Study of Paricalcitol plus Nanoliposomal Irinotecan and 5-FU/LV in Advanced Pancreatic Cancer Patients after Progression on Gemcitabine-Based Therapy. Clin. Cancer Res..

[132] Wu X., Hu W., Lu L., Zhao Y., Zhou Y., Xiao Z., Zhang L., Zhang H., Li X., Li W. (2019). Repurposing Vitamin D for Treatment of Human Malignancies via Targeting Tumor Microenvironment. Acta Pharm. Sin. B.

